# Targeted High Resolution LC/MS^3^ Adductomics Method for the Characterization of Endogenous DNA Damage

**DOI:** 10.3389/fchem.2019.00658

**Published:** 2019-10-24

**Authors:** Andrea Carrà, Valeria Guidolin, Romel P. Dator, Pramod Upadhyaya, Fekadu Kassie, Peter W. Villalta, Silvia Balbo

**Affiliations:** Masonic Cancer Center, University of Minnesota, Minneapolis, MN, United States

**Keywords:** mass spectrometry, DNA adductomics, DNA damage, cancer, inflammation, lipid peroxidation, tobacco carcinogens

## Abstract

DNA can be damaged through covalent modifications of the nucleobases by endogenous processes. These modifications, commonly referred to as DNA adducts, can persist and may lead to mutations, and ultimately to the initiation of cancer. A screening methodology for the majority of known endogenous DNA adducts would be a powerful tool for investigating the etiology of cancer and for the identification of individuals at high-risk to the detrimental effects of DNA damage. This idea led to the development of a DNA adductomic approach using high resolution data-dependent scanning, an extensive MS^2^ fragmentation inclusion list of known endogenous adducts, and neutral loss MS^3^ triggering to profile all DNA modifications. In this method, the detection of endogenous DNA adducts is performed by observation of their corresponding MS^3^ neutral loss triggered events and their relative quantitation using the corresponding full scan extracted ion chromatograms. The method's inclusion list consists of the majority of known endogenous DNA adducts, compiled, and reported here, as well as adducts specific to tobacco exposure included to compare the performance of the method with previously developed targeted approaches. The sensitivity of the method was maximized by reduction of extraneous background signal through the purification and minimization of the amount of commercially obtained enzymes used for the DNA hydrolysis. In addition, post-hydrolysis sample purification was performed using off-line HPLC fraction collection to eliminate the highly abundant unmodified bases, and to avoid introduction of plasticizers found in solid-phase extraction cartridges. Also, several instrument parameters were evaluated to optimize the ion signal intensities and fragmentation spectra quality. The method was tested on an animal model of lung carcinogenesis where A/J mice were exposed to the tobacco specific lung carcinogen 4-methylnitrosamino-1-3-pyridyl-1-butanone (NNK) with its effects enhanced by co-exposure to the pro-inflammatory agent lipopolysaccharide (LPS). Lung DNA were screened for endogenous DNA adducts known to result from oxidative stress and LPS-induced lipid peroxidation, as well as for adducts due to NNK exposure. The relative quantitation of the detected DNA adducts was performed using parallel reaction monitoring MS^2^ analysis, demonstrating a general workflow for analysis of endogenous DNA adducts.

## Introduction

Covalent modifications of DNA, commonly referred to as DNA adducts, occur extensively due to both endogenous processes and exogenous exposures (Tretyakova et al., 2013). An extensive enzymatic repair system exists to eliminate adducts, however if adduct formation persists it can lead to genomic instability and may result in mutations of the normal DNA sequence (Shrivastav et al., 2010). The persistence of these mutations can result in altered gene expression, abnormal cell growth, disruption of normal cellular function, and ultimately to the initiation of cancer (Delaney and Essigmann, 2008; Loeb and Harris, 2008). Therefore, measurement of these DNA adducts is critical to understanding cancer etiology and to assess the carcinogenic effects of specific exposures, ultimately to allow the identification of their mechanism of action and potentially recognize individuals at higher cancer risk.

Genotoxicity testing, including assessment of DNA adduct formation, is widely used to investigate the potential carcinogenic effects of specific exposures, such as substances used in industrial and manufacturers' processes, environmental pollutants, and to life-style factors associated with increased cancer risk (Food and Drug Administration HHS, 2012). Important analytical limitations hamper the application of currently used methods to screen for DNA adducts in humans. Methods such as ^32^P-postlabelling or the Comet Assay, lack the specificity and selectivity to identify specific DNA adducts, while traditional LC-MS^2^-based approaches are restricted to analyzing only a few modifications at a time (Beach and Gupta, 1992; Tretyakova et al., 2012, 2013).

DNA adductomics, a methodology currently under development for screening and identification of DNA adducts, can be sensitive, specific, selective, and comprehensive (Balbo et al., 2014b; Villalta and Balbo, 2017). Initial DNA adductomics investigations typically used neutral loss or “pseudo-neutral loss” scanning with “low resolution” triple quadrupole and ion trap instrumentation, but has more recently been performed with high resolution instruments such as Q-TOFs and Orbitraps, increasing the sophistication and power of the basic methodology (Balbo et al., 2014b; Villalta and Balbo, 2017). In particular, the use of multistage scanning (MS^n^) and high resolution accurate mass (HRAM) data acquisition offers the possibility of unambiguous identification and chemical characterization of many DNA adducts simultaneously. We have previously developed a Data-Dependent Constant Neutral Loss-Triple Stage Mass Spectrometry (DDA-CNL/MS^3^) methodology, similar to an earlier ion trap-based approach, using high resolution accurate mass detection and monitoring of the exact mass neutral loss of deoxyribose (dR = 116.0474 ± 0.0006 *m/z*) to increase specificity, and provide for molecular formula determination of the adduct and MS^2^ and MS^3^ fragment ions (Balbo et al., 2014a; Stornetta et al., 2015). The power of this approach was demonstrated in the analysis of DNA adducts in lung tissue of mice treated with NNK, simultaneously detecting multiple DNA modifications and gaining structural information of both expected and unknown DNA adducts (Balbo et al., 2014a). This proof-of-principle study required fractionation and multiple injections per fraction to provide sufficient sensitivity, therefore limiting its usefulness (Balbo et al., 2014a). A modification of the approach using a large inclusion list of likely mono- and cross-linked DNA adducts was applied to characterize the DNA adducts from cells exposed to a chemotherapeutic agent (Stornetta et al., 2015). In addition, the neutral loss of the DNA bases in the MS^3^ triggering step allowed detection of those adducts for which the base-deoxyribose bond is unstable (Stornetta et al., 2015). Although the use of the inclusion list allowed characterization of DNA adducts in a single LC/MS run, the adducts of interest were relatively large and hydrophobic (Stornetta et al., 2015). Therefore, improvements to the DNA adductomic approach are needed to allow comprehensive characterization of the majority of known endogenous DNA adducts, including hydrophobic and hydrophilic adducts of various sizes, in *in vivo* samples with a single injection.

Our long-term goal is to translate the DDA-CNL/MS^3^ approach described above into a routine, high-throughput method that will allow for the detection, and relative quantitation of the majority of known endogenous DNA adducts and exposure-specific adducts. This task is challenging as most endogenous DNA adducts are hydrophilic, and are similar in mass and chromatographic behavior to the unmodified nucleosides and ubiquitous background signal (generally 200–350 *m/z*) (Guo et al., 2017). In the present study, a database of known endogenous DNA adducts has been created and used to populate an inclusion list for MS^2^ selection, thereby greatly improving the specificity of the MS^2^ fragmentation events. In addition, an off-line sample fractionation approach was developed whereby the narrow fractions containing the unmodified nucleosides were discarded and the rest of the fractions were combined into a single sample for LC-MS analysis. The source of background ion signal, which greatly reduces the overall sensitivity of the approach, was investigated. Furthermore, the method was transferred from an ion trap-Orbitrap hybrid instrument (Orbitrap Velos) to a more sensitive and powerful quadrupole-ion trap-high field Orbitrap instrument (Orbitrap Fusion). The analytical parameters were carefully optimized for the new platform using a standard mix of various adducted DNA nucleosides with varying size and hydrophobicity. The new DNA adductomic approach was then tested by analyzing lung DNA from A/J mice treated with 4-(methylnitrosamino)-1-(3-pyridyl)-1-butanone (NNK) and lipopolysaccharide (LPS) to gain insights into the role of inflammation in the development of lung cancer.

## Materials and Methods

### Chemicals

Methanol (LC-MS grade), acetonitrile (LC-MS grade), isopropanol (IPA), formic acid (FA, 98% _v/v_) were purchased from Fluka (St. Louis, MO, USA). 4-(methylnitrosamino)-1-(3-pyridyl)-1-butanone (NNK; 98%), lipopolysaccharide (LPS) from *Escherichia coli*, and NaBH_3_CN were purchased from Sigma (St. Louis, MO, USA). Distilled water was purified by a Milli-Q system (Milford, MA, USA). Deoxyribonuclease I from bovine pancreas (DNase, 2,000 U/mg), DNase I recombinant expressed by *Pichia pastoris* (R-DNase, 10,000 U/mg), phosphodiesterase-1 extracted from *Crotalus adamanteus* (PDE-1, 0.4 U/mg), alkaline phosphatase extracted from calf intestine (ALP, 3,000 U/mg), recombinant alkaline phosphatase highly active expressed by *Pichia pastoris* (R-ALP, 7,000 U/mg), and calf thymus DNA (5 mg) were purchased from Roche (St. Louis, MO, USA). Double filtration membrane Amicon Ultra (30 kDa cutoff, 0.5 mL) and single filtration membrane Microcon (10 kDa cutoff, 0.5 mL) were purchased from Amicon (Billerica, MA, USA). Silanized vials (0.3 mL, 1.5 mL and 4 mL) were purchased from ChromTech (Apple Valley, MN, USA). *N*^2^-ethyl-2′deoxyguanosine (*N*^2^-ethyl-dG), (6R/S)-3-(2′-deoxyribos-1′-yl)-5,6,7,8-tetrahydro-6-pyrimido[1,2-a]-purine-10(3H)one (pro-dG), *O*^6^-[4-(3-pyridyl)-4-oxobut-1-yl]-2′-deoxyguanosine (*O*^6^-POB-dG), *O*^6^-[4-(3-pyridyl)-4-oxobut-1-yl]-2′-deoxyguanosine (*O*^6^-POB-dG), *O*^2^-[4-(3-pyridyl)-4-oxobut-1-yl]thymidine (*O*^2^-POB-dT), *O*^6^-methyl-2′-deoxyguanosine (*O*^6^-methyl-dG), and *N*^6^-methyl- deoxyadenosine (*N*^6^-methyl-dA) were synthesized as described by Wan et al. (2005). 6-(1-Hydroxyhexanyl)-8-hydroxy-1, *N*^2^-propano-2-deoxyguanosine (HNE-dG) was a kind gift from Dr. Fung-Lung Chung of Georgetown University.

### Treatment of Mice With NNK and LPS

Female A/J mice, 6 weeks of age, were purchased from The Jackson Laboratory (Bar Harbor, ME, USA). Upon arrival, the mice were housed in specific pathogen-free animal quarters at the Research Animal Resources, University of Minnesota Academic Health Center. Handling and treatment was in accordance with an animal study protocol approved by the Institutional Animal Care and Use Committee (Protocol ID: 1602-33469A). Mice were treated with NNK and LPS, following the study design previously reported and briefly summarized in Table S1 (Melkamu et al., 2013; Song et al., 2015). NNK (100 mg/kg) and LPS (5 μg/day) were administered via intraperitoneal injection (IP) and intranasal instillation, respectively. The mice were euthanized by an overdose of carbon dioxide on the fourth day from exposure. Subsequently, lung tissues were harvested and stored at −80°C until analysis.

### DNA Isolation From Mouse Lung

DNA isolation was performed following a previously reported method (Balbo et al., 2008). Briefly, 50 mg of lung tissue were homogenized in 1.5 mL of cell lysis solution (Qiagen). The homogenization was performed using a tissue rupture homogenizer (Qiagen). Samples were then treated with Proteinase-K (24 h, RT) and RNase-A (2 h, RT). Proteins were precipitated by adding 0.5 mL of Protein Precipitation Solution (Qiagen). The pellets were discarded and the supernatant was transferred into a new tube containing an equal amount of cold IPA to precipitate the DNA. The solid DNA was manually extracted from the IPA and dissolved in Tris/EDTA (10 mM, 5 Mm, pH 7) aqueous buffer. Lipids and hydrophobic impurities were excluded by liquid-liquid extraction using chloroform and isoamyl alcohol (24:1_v/v_, 0.5 mL). The mixture was centrifuged and the upper aqueous layer containing the DNA was carefully transferred into a new tube containing cold IPA (0.5 mL) to precipitate the DNA. The mixture was centrifuged and the supernatant was discarded and the DNA pellet washed with 0.5 mL of IPA 70%_v/v_ in water and then with 100% IPA. The pellets were dried, resuspended in Tris buffer, and stored at −20°C. The yield and purity of the DNA was assessed using a nanodrop UV/Vis spectrophotometer monitoring the 260 nm and the 280 nm wavelengths (Zimmer and Roalson, 2005). The extraction yields typically ranged from 2 to 5 μg of DNA per 1 mg of sample.

### DNA Stabilization and Digestion

Many endogenously formed DNA adducts result from the reaction of an aldehydic moiety with the exocyclic amino groups of the nucleotides. This results in Schiff bases that are stable in the intact DNA helix but easily degrade upon DNA hydrolysis. The use of NaBH_3_CN, a mild reducing agent, stabilizes the adducts and allows for their LC-MS analysis (Wang et al., 2000). Two different strategies were evaluated for the reduction and stabilization of the Schiff base adducts and the subsequent enzymatic digestion of the DNA. In the first protocol, the amounts of the hydrolysis enzymes were increased in order to compensate for their deactivation in the presence of the NaBH_3_CN (DNA digestion protocol 1). Approximately 50 μg of DNA was digested in the presence of 30 mg of NaBH_3_CN, 2000 U of DNase, 80 mU of PDE-1 and 1000 U of ALP. In the second protocol, the DNA-stabilization and the enzymatic digestion were carried out in two different reaction vessels, avoiding the direct interaction of the enzymes and NaBH_3_CN (DNA digestion protocol 2). After NaBH_3_CN treatment (30 mg), the DNA was isolated and desalted with a double filtration membrane (Amicon Ultra 0.5 mL, 30 kDa cutoff), and washed with 1.2 mL of Tris buffer. The desalted DNA was reconstituted with 300 μL of Tris and transferred from the filtration membrane into a clean reaction vial. The DNA recovery from the filtration membrane was assessed using a UV/Vis spectrophotometer (typical recovery 75–90%). The enzymatic digestion was then performed by incubating the DNA (approximately 50 μg) with 50 U of DNase, 2 mU of PDE-1 and 20 U of ALP at RT overnight. After digestion, the enzymes were removed by using a filtration membrane (Amicon Ultra 0.5 mL, 10 kDa cutoff). Table S2 summarizes the experimental conditions of the two digestion protocols. The digestion yields were assessed by quantifying deoxyguanosine (dG) by UPLC with UV detection (Ultimate 3000, Thermo Scientific, Waltham, MA). The chromatographic conditions and the elution program are reported in the Supplemental Information along with a typical chromatogram (Figure S1) and calibration curve for dG quantitation (Figure S2). The recovery of the DNA adducts was determined by spiking the samples with a mixture of isotopically labeled internal standards ([^15^N_5_]-*N*^2^-ethyl-dG, [^15^N_5_]-*N*^6^-methyl-dA and R,S-[^15^N_5_]-pro-dG, 100 fmol each).

### Sample Purification and Enrichment

Sample purification after enzymatic digestion was performed using HPLC fractionation to remove the unmodified nucleosides (dRNs) from the sample. Fractionation was carried out with an HPLC (Ultimate 3000, Thermo Scientific, Waltham, MA) equipped with a C18 column (4.6 × 250 mm, 100 Å, 5 μm Luna Phenomenex, Torrance, CA) operated at 5°C, with a flow rate of 1.0 mL/min. The mobile phases A and B consisted of water (H_2_O) and methanol (MeOH), respectively. The elution program involved an isocratic step at 2% of B (5 min), followed by a linear gradient of 0.7% B/min (25 min) and a second isocratic step at 100% of B (15 min). At the end of the elution, the LC-system was equilibrated in isocratic conditions (2% of B) for 20 min. The UV detector was operated at 4 Hz in absorbance-mode, probing two different wavelengths (λ^1^ 190 nm and λ^2^ 254 nm) for monitoring the elution of the dRNs. Ten different fractions were collected: fraction-0 (Fr0, 0–15 min), fraction-1 (Fr1, 15–20.5 min), fraction-dC (Fr-dC, 20.5–22.5 min), fraction-2 (Fr2, 22.5–26.5 min), fraction-dG-dT (Fr-dT, 26.5–28 min), fraction-3 (Fr3, 28–31 min), fraction-dA (Fr-dA, 31–32 min), fraction-4 (Fr4, 32–39 min), fraction-5 (Fr5, 39–46 min), and fraction-6 (Fr6, 46–53.00). A representative HPLC-UV chromatogram with gradient and fraction collection information is shown in Figure S3. After each fractionation, the collected fractions (Fr1-6), which did not contain the unmodified dRNs, were combined along with 100 fmol of [D_4_]-*O*^6^-POB-dG, dried and frozen until LC-MS analysis. The fractions containing the unmodified dRNs (Fr-dC, Fr-dG, Fr-dT, and Fr-dA) were discarded.

### Signal Optimization

Analyte dependent instrumental parameters (e.g., S-Lens, collision induced dissociation (CID), high-energy collisional dissociation (HCD), etc.), were optimized by infusion of a synthetic standard mixture of representative DNA adducts (*N*^6^-methyl-dA, *O*^6^-methyl-dG, *N*^2^-ethyl-dG, pro-dG, and HNE-dG) directly into the ion source. Parameters were varied and signal intensity measured. The molecular structure of these DNA adducts and their characteristic fragmentation ions are reported in Figure S4.

### LC Conditions for MS Analysis

The dried hydrolyzed DNA samples were brought to RT in 20 min, reconstituted in 20 μL of water (LC-MS grade, Fluka) and then analyzed by LC-MS. For each sample 3 μL were injected. The LC was performed using a nanoflow UPLC (Ultimate 3000 RSLCnano UPLC, Thermo Scientific, Waltham, MA). The UPLC was equipped with a 5 μL loop and reversed-phase chromatographic separation was performed using a hand-packed commercially available fused-silica emitter (230 × 0.075 mm ID, 15 μm orifice, New Objective, Woburn MA) with C18 stationary phase (5 μm, 100, Luna Phenomenex, Torrance, CA). The mobile phase consisted of an aqueous solution of 0.05 %_v/v_ formic acid (phase-A) and CH_3_CN (phase-B). The elution program included an isocratic step (2 % of B for 5 min at 1 μL/min), followed by a linear gradient of B (1.5%/min for 25 min at 0.3 μL/min) and it concluded with a washing isocratic step, performed at 98% of B for 5 min at 0.3 μL/min. At the end of the elution program, the LC-system was equilibrated for 5 min at isocratic condition (2% of B, 1 μL/min). At 6 min., the injection valve was switched to remove the sample loop from the liquid flow path. The injection valve and needle were washed with 200 μl of acetonitrile between sample injections to avoid carryover.

### Mass Spectrometry

All mass spectrometry was performed using a hybrid high-field Orbitrap mass spectrometer (Fusion, Thermo Scientific, Waltham, MA). The LC system was interfaced to the mass spectrometer using a Nanoflex ESI ion source (Nanoflex Thermo Scientific, Waltham, MA), which operated in positive ion mode at 2.5 kV. the transfer ion tube temperature was 350°C, and S-Lens setting was 60.

#### Targeted Adductomic Screening Analysis

The screening method was performed using DDA-CNL/MS^3^ analysis that consists of three detection events: full scan, targeted data dependent MS^2^ acquisition (MS^2^) and a neutral loss MS^3^ data acquisition (NL-MS^3^). The full scan (100–1,000 *m/z*) was performed with quadrupole filtering, maximum injection time of 50 ms, automatic gain control (AGC) of 5 × 10^4^, and a resolution setting of 60,000. An inclusion list of 128 DNA adducts (Table S3, https://docs.google.com/spreadsheets/d/117ZmOIfToQuvzf_4g4kvi0ZESjMqAhsM/edit#gid=413192527) was used to trigger MS^2^ scan events, with a mass tolerance of 5 ppm, dynamic exclusion of 20 s, intensity threshold of 10^4^ counts, and a quadrupole isolation width of 1.5 *m/z*. The MS^2^ fragmentation was performed in the linear ion trap (LIT) with a CID collision energy of 30% and an activation time of 10 ms, with Orbitrap detection at a resolution of 15,000 and a maximum injection time of 200 ms. For the NL-MS^3^ data acquisitions, MS^2^ product ions were isolated in the ion trap with an isolation width of 3 *m/z*, and MS^3^ fragmentation triggered upon observation of the neutral loss of the dR moiety (-dR; 116.0474 ± 0.0006 *m/z*, ± 5 ppm), with HCD fragmentation (50%), and Orbitrap detection at a resolution of 15,000 and a maximum injection time of 300 ms. LC and injection conditions are as reported in the LC Conditions for MS Analysis section.

#### Targeted Adduct Analysis

The targeted adduct analysis was performed using PRM-MS^2^ (Villalta et al., 2017) of 73 ions (4 internal standards, 69 DNA adducts) with quadrupole isolation widths of 2 *m/z*, maximum injection times of 22 ms, AGC of 5 × 10^4^, and resolution setting of 15,000. Fragmentation was performed using HCD with a collision energy of 30%. The fragmentation of the DNA adduct precursor ions [MH]^+^ results in neutral loss of the dR moiety to produce the corresponding [MH-116.0474]^+^ product ions whose masses are extracted from the PRM signal for detection of the adducts. LC and injection conditions are as reported in LC Conditions for MS Analysis section.

## Results

### Generation of Endogenous DNA Adduct Database

A database of endogenous DNA adducts consisting of adducts reported in the literature including those from alkylation, lipid peroxidation (LPO) and from reactive oxygen species (ROS) (Table S3) was created, and used to generate the inclusion list for our adductomic method. The database includes the name, chemical formula, [M+H]^+^ *m/z*, origin, literature reference and chemical structure and is available at https://drive.google.com/open?id=14r9mA8NlL908piFCLA5yP-BZAsilxUl7.

### Optimization of Instrumental Parameters

**Figure 1:**
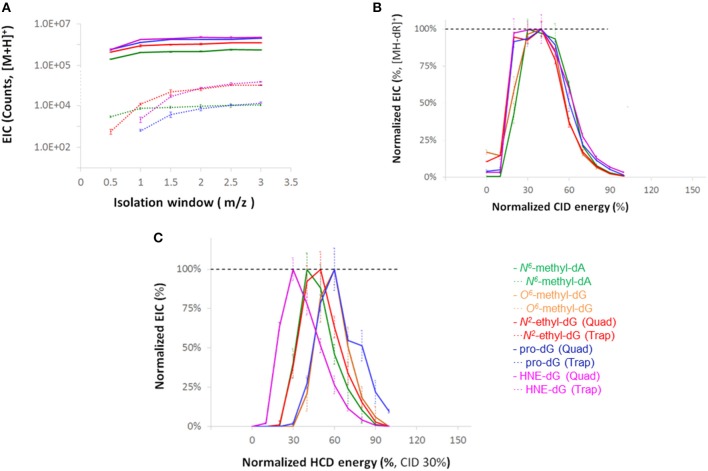
**(A)** Extracted ion chromatogram (EIC) signal intensity for the four adducts with quadrupole and ion trap isolation as a function of isolation width. **(B)** Intensities of MS^2^ product ions resulting from neutral loss of dR moiety **(C)** Intensities of base peak ions for MS^3^ HCD fragmentation with various collision energies where the [MH-dR]^+^ ions were generated and isolated in the linear trap, at a CID-level of 30% and isolation width of 3.0 *m/z*.

Optimization of instrumental parameters was carried out through infusion of a diverse set of DNA adducts (Figure S4) at a concentration of 0.5 pmol/μL (1:1 H_2_O/MeOH, FA 0.1 %) and a flow rate of 5 μL/min. The full scan ion signals of the adducts as a function of S-Lens setting are shown in Figure S5. MS^2^ and NL-MS^3^ data acquisition parameters were optimized by measuring signal intensities as a function of the CID and HCD collision energies, and quadrupole and linear ion trap isolation widths and summarized in Figure 1. The relative isolation efficiencies of the quadrupole and linear ion trap are illustrated in Panel A. The normalized ion signals of the fragment ions [MH-dR]^+^ plotted as a function of CID collision energy are shown in Panel B. The range of optimal MS^3^ collision energies in terms of maximum base peak intensities are summarized in Panel C.

### Minimization of Background Ion Signal

Sources of background ion signals were evaluated using the DNA digestion protocol 2 conditions reported in DNA Stabilization and Digestion Section. Four aliquots of Tris buffer (300 μL) were spiked with one of the matrix component: DNA (50 μg), DNase (50 U), PDE-1 (2.0 mU), and ALP (20 U). Along with them, a DNA sample (50 μg) was also prepared and digested, purified following the fraction collection procedure, dried, reconstituted with 20 μL of Milli-Q H_2_O and analyzed using LC-MS. A visual comparison of the TIC chromatograms is reported in the Figure S6. Some impurities were common to all samples (e.g., PEG-400, [M+H]^+^ 428 *m/z*, red asterisks [^*^]), whereas others were specific to the DNA digested samples (e.g., dA, [M+H]^+^ 252 *m/z*, green spot [•]). In the DNA Stabilization and Digestion Section, it was determined (Figure S1) that the presence of NaBH_3_CN adversely affected the enzyme activity and necessitated the use of much higher amounts of enzymes than used here to assure complete DNA hydrolysis, and this would result in significantly higher background ion signals than those reported in Figure S6.

### Ion Suppression

The efficiency of analyte ionization can be adversely effected by the presence of sample matrix material by a phenomenon known as ion suppression (Annesley, 2003). To assess the extent of ion suppression in the DNA hydrolysis samples, aliquots of calf-thymus DNA at amounts similar to those used in the analysis were processed according to the standard digestion and purification protocols. The collected fractions were dried and reconstituted with 20 μL of a 5 fmol/μL solution of isotopically labeled synthetic standards **([**^15^N_5_]-*N*^6^-methyl-dA, **[**^15^N_5_]-*N*^2^-ethyl-dG, **[**^15^N_5_]-pro-dG, [D_4_]-*O*^2^-POB-dT, and [D_4_]-*O*^6^-POB-dG). These samples were analyzed using the LC-MS methodology and the ion suppression was calculated by comparison of the precursor ion signals of the standards of neat solutions to those spiked into DNA samples after sample preparation (Table 1) (Furey et al., 2013). These results indicate minimal ion suppression for the analytes tested.

**Table 1:** Chromatographic peak areas of the isotopically labeled standards in the neat sample and triplicate set of spiked DNA samples (final concentrations of 5 fmol/μL), and the calculated values of ion suppression.

	**Area (Ion suppression)**			
	**Standard mix**	**DNA sample 1**	**DNA sample 2**	**DNA sample 3**
[^15^N_5_]-*N^6^*-methyl-dA	4.75 × 10^7^	5.08 × 10^7^ (−7%)	4.79 × 10^7^ (−1%)	4.95 × 10^7^ (−4%)
[^15^N_5_]-*N^2^*-ethyl-dG	1.62 × 10^7^	1.53 × 10^7^ (6%)	1.83 × 10^7^ (−13%)	1.96 × 10^7^ (−21%)
[^15^N_5_]-pro-dG	1.90 × 10^8^	2.05 × 10^8^ (−8%)	1.91 × 10^8^ (0%)	1.83 × 10^8^ (4%)
[D_4_]-*O^2^*-POB-dT	2.44 × 10^7^	3.10 × 10^7^ (−27%)	2.37 × 10^7^ (3%)	2.54 × 10^7^ (−4%)
[D_4_]-*O^6^*-POB-dG	4.46 × 10^7^	4.35 × 10^7^ (−27%)	6.17 × 10^7^ (3%)	5.49 × 10^7^ (−4%)

### Recovery

The recovery for the method was assessed by generating 2 sets of DNA samples in triplicate using the digestion and fraction collection procedure discussed here, with addition of 100 fmol of isotopically labeled internal standards (**[**^15^N_5_]–*N*^6^-methyl-dA, **[**^15^N_5_]-*N*^2^-ethyl-dG, **[**^15^N_5_]-pro-dG, [D_4_]-*O*^2^-POB-dT, and [D_4_]-*O*^6^-POB-dG) prior to enzymatic digestion for one set and reconstituted with the same standard mixture for a second set. The recovery was calculated by comparing the chromatographic peak area of the standards in the two sample sets (Table 2), and found to range from 28 to 59% while the reproducibilities ranged from 4 to 14% (±1 STD). The experimental reproducibility was also assessed by comparing the extracted-ion chromatogram (EIC) areas and reproducibility expressed as % coefficient of variation (CV): [^15^N_5_]-*N*^6^-methyl-dA (25%), [^15^N_5_]-*N*^2^-ethyl-dG (25%), [^15^N_5_]-pro-dG (18%), [D_4_]-*O*^2^-POB-dT (23%), and [D_4_]-*O*^6^-POB-dG (8%). The instrumental repeatability was also assessed by injection of a mixture of standards (2.5 fmol/μL) before each experiment and the instrumental response was found to have % CV <10% (SI, Table S4).

**Table 2:** Assessment of analyte recoveries: Group 1 samples spiked with internal standards (100 fmol) at the beginning of the enzymatic digestion. Group 2 samples spiked with internal standards (100 fmol) at the end of the sample preparation.

	**Group 1**	**Group 2**	**Recovery**
	**Ave. ± STD (× 10^**7**^)**	**Ave. ± STD (× 10^**7**^)**	**Ave. ± STD**
[^15^N_5_]-*N^6^*-methyl-dA	0.77 ± 0.19	1.78 ± 0.22	42 ± 11%
[^15^N_5_]-*N^2^*- ethyl-dG	2.2 ± 0.55	4.94 ± 0.15	48 ± 11%
[^15^N_5_]-pro-dG	5.6 ± 0.99	19.3 ± 1.1	28 ± 5%
[D_4_]-*O^2^*-POB-dT	1.6 ± 0.37	2.67 ± 0.38	59 ± 14%
[D_4_]-*O^6^*-POB-dG	3.0 ± 0.23	5.34 ± 0.92	55 ± 4%

### Sensitivity, Linearity, and Limits of Detection

Sensitivity and detection limits were assessed by spiking the standard mixture at different concentrations into processed DNA samples. The DNA samples were prepared by hydrolyzing 250 μg of DNA, followed by purification via off-line fraction collection, and combination of the collected fractions into a single sample. The DNA solution was then divided into five aliquots (equivalent to 50 μg DNA each), dried, and reconstituted using the isotopically labeled standard mixture (0.0, 0.2, 1.0, 2.0, 5.0 fmol/μL of [^15^N_5_]-*N*^6^-methyl-dA, [^15^N_5_]-*N*^2^-ethyl-dG, [^15^N_5_]-pro-dG, [D_4_]-*O*^2^-POB-dT, and [D_4_]-*O*^6^-POB-dG). The calibration curves were obtained by plotting the EIC peak area of the standards from the full scan mass spectral data with a mass tolerance of ± 5 ppm (Figure S7). Similarly, calibration curves were also generated for the MS^2^ detection event by extracting the signal of [MH-dR]^+^ molecular ions from the full MS^2^ TIC spectra. The linearity of the response of the method for the various adduct standards was calculated as the slope and coefficient of determination (R^2^) values of the calibration curves. The limits of detection (LODs) were calculated as the amount of analytes required to generate [MH-dR]^+^ ions, whose signals were three-fold greater than the threshold limit imposed in the method (5 × 10^3^ counts for the MS^2^). This unconventional definition was used because the MS^3^ data acquisition is the detection event of the adductomic approach, thus the overall sensitivity reflects the ability of the instrument to trigger MS^3^ detection events. The linearity of the instrumental response, the sensitivity and the limits of detection in the full scan and MS^2^ data acquisitions are reported in Table 3.

**Table 3:** Assessment of instrumental sensitivity, linearity, and detection limits.

**Detection of [M+H]**^****+****^ **full scan data acquisition**				
	**Signal intensity** **(× 10**^**5**^ **cts/**^**.**^**fmol on-column)**	Coefficient of determination^*^ **(*****R***^**2**^**)**	**LOD** **(fmol/μmol dG)**	**Adducts (per 10**^**8**^ **nucleotides)**
[^15^N_5_]-*N^6^*-methyl-dA	1.3	0.99	28	7
[^15^N_5_]-*N^2^*-ethyl-dG	3.7	0.99	10	2
[^15^N_5_]-pro-dG	0.4	0.98	88	0.2
[D_4_]-*O^2^*-POB-dT	3.0	0.99	12	3
[D_4_]-*O^6^*-POB-dG	2.6	0.98	14	3

* *Linearity was measure over the 0.6–15 fmol (on-column) range*.

### A/J Mice Treated With LPS/NNK: DNA Adduct Detection and Profiling

Lung tissues were obtained from four mice (no treatment, NNK only, LPS only, and NNK and LPS). Experimental details are described above. DNA was extracted from the tissues, hydrolyzed and purified as described in the method section. A negative control was generated by processing Tris buffer with no DNA using the same work up as used for processing the DNA samples. The complexity of the DNA samples (6 × 10^6^ full scan ions detected) and the ability of the method to screen for trace level DNA adducts is demonstrated by the resulting 4,500 MS^2^ fragmentation events and 250 MS^3^ spectra as shown in Figure S8. The samples were analyzed using the DDA-CNL/MS^3^ analysis and adducts were identified by considering all the masses characterized by the presence of the MS^3^ signal. It was verified that each MS^3^ signal corresponded to a parent ion in the inclusion list with a clear signal in the full scan event, and excluding any of the ions that were present as full scan EIC peaks in the negative control at a similar retention time.

**Figure 2:**
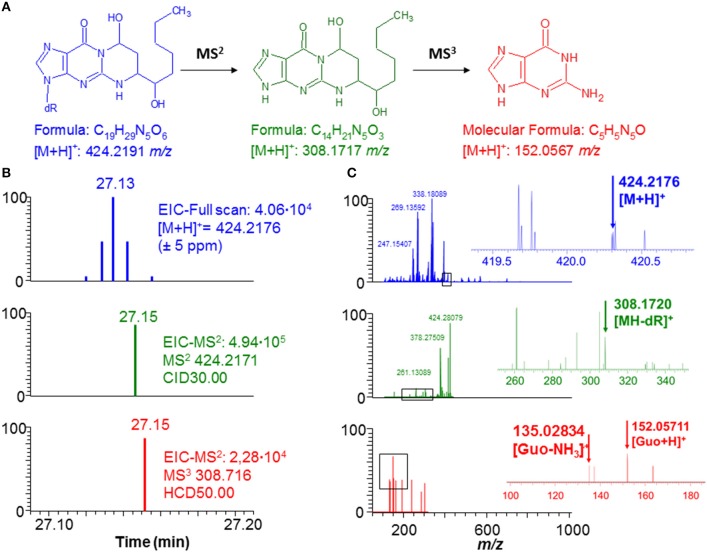
Typical example of DNA adduct (HNE-dG) detected in a lung sample treated with LPS. **(A)** Fragmentation pathway of the DNA adduct (HNE-dG) ion. **(B)** The scan events corresponding to the full scan (blue), MS^2^ (green), and MS^3^ (red) spectra indicating the presence of the HNE-dG ion. **(C)** The spectra corresponding to the full scan (blue), MS^2^ (green), MS^3^ (red) scan events shown in **(B)**.

DNA adduct identifications were confirmed, when possible, by spiking with stable isotopically labeled internal standards or by comparison of the MS^2^ and MS^3^ fragmentation spectra and retention time with that of unlabeled synthetic standards. Figure 2 shows an example of DNA adduct fragmentation and characterization. HNE-dG was detected in the LPS-exposed sample. The identity of the putative adduct was subsequently confirmed by comparison of retention time, and MS^2^ and MS^3^ fragmentation spectra with a HNE-dG (2.5 fmol/μL) synthetic standard. The expected NNK-derived DNA adducts *O*^2^-PHB-dT and *O*^6^-methyl-dG were also confirmed in a similar fashion in both NNK and LPS/NNK treated mice, respectively. In addition, the detection of constitutional isomers of endogenous and exogenous DNA adducts in the inclusion list was evaluated (Figure S9).

**Figure 3:**
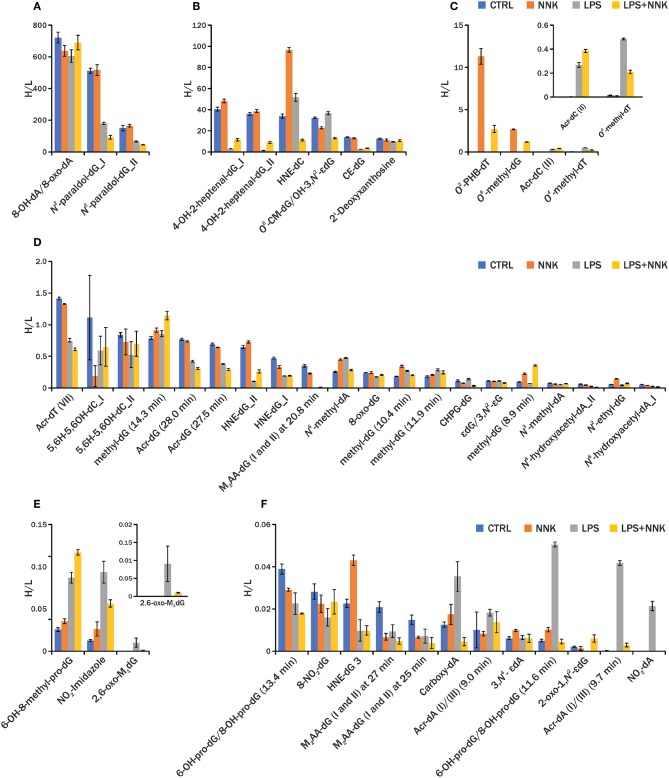
Relative quantification of DNA adducts among different DNA samples **(A–F)**. Blue, orange, gray, and yellow histograms are levels of DNA adducts detected in lung sample from controls and animals treated with NNK, LPS and LPS+NNK, respectively. Reported here is the AUC/ [^15^N_5_]-*N*^2^-ethyl-dG H/L for each DNA adduct in different groups of samples (*n* = 3). Retention times are reported for the DNA adducts that were detected as multiple isomers.

**Figure 4:**
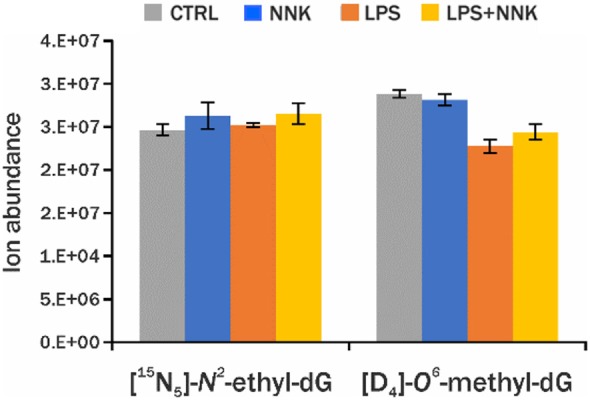
Blue, orange, gray, and yellow histograms are levels of DNA adducts internal standards ([^5^N_5_]-*N*^2^-ethyl-dG, [D_4_]-*O*^6^-methyl-dG) detected in lung sample from controls and animals treated with NNK, LPS, and LPS+NNK, respectively.

The comparison of the detected adduct level across samples within an experiment is critical to understand the DNA damage being investigated. The traditional LC-MS methods used to quantify specific DNA adducts rely on the use of isotopically labeled standards, however it is not practical, or even currently possible, to include all internal standards necessary for screening of all DNA adducts in our endogenous DNA adduct database. For this reason, relative quantitation was performed using PRM-MS^2^ analysis of all adducts detected using DDA-CNL/MS^3^ analysis by generating EICs of the corresponding [MH-dR]^+^ ions and measuring the area under the curve (AUC) of the chromatographic peak (Higgs et al., 2005). This analysis was performed on a second group of samples (one NNK, one LPS+NNK, one LPS and one control), to relatively quantify the adducts and to evaluate the instrumental repeatability of the method in *in vivo* samples. The AUCs of the 36 DNA adducts were normalized by the AUC of the [^15^N_5_]-*N*^2^-ethyl-dG internal standard, spiked into the sample for quantification, and to the amount of DNA digested. Three injections were made from each processed sample to provide technical replicates to check instrumental precision of the relative quantitative values, and low standard deviations for the AUCs of the EICs of all DNA adducts measured (Figure 3) including the internal standards (CV = 5% for [^15^N_5_]-*N*^2^-ethyl-dG and CV = 11% for [D_4_]-*O*^6^-methyl-dG, Figure 4) were observed.

In general, data show that the adductomic based method was able to profile both DNA adducts generated endogenously by lipid peroxidation (LPO) and reactive oxygen species (ROS) due to LPS exposure and by NNK exposure in *in vivo* samples (Table S5). In addition, the data demonstrated that the method was able to putatively assign a chemical structures to detected DNA adducts on the basis of the accurate mass, neutral loss and MS^3^ fragmentation spectra (Figure 2). The detection of unknown constitutional isomers of endogenous and exogenous DNA adducts in the inclusion list is also possible with the method. An example is shown in Figure S9 demonstrating the presence of *O*^6^-methyl-dG with the various isomers. 36 DNA adducts (including 2 labeled internal standard adducts) detected in the DDA-CNL/MS^3^ screening assay were targeted for relative quantitation using the PRM-MS^2^ assay in a second group of mice lung tissue samples (one NNK, one LPS+NNK, one LPS and one control). The results demonstrate the successful use of an analytical workflow for the identification of endogenous DNA present in a sample type using DDA-CNL/MS^3^ and subsequent relative quantitation of the found adducts using PRM-MS^2^. Only instrumental technical replicates were performed and therefore no conclusion on the toxicological and biological relevance of DNA adduct levels measured in the four treatment groups could be made. DNA modifications related to oxidative stress (e.g., NO_2_-dA) and lipid peroxidation (e.g., M_1_-dG) were observed in the LPS treated samples, whereas PHB DNA adducts and *O*^6^-methyl-dG were detected in all the samples exposed to NNK (e.g., *O*^2^-PHB-dT) (Figure 3) (Ayala et al., 2014). These results confirmed the ability of the methods to reveal DNA damages specifically induced by each individual treatment.

## Discussion

DNA adductomic analysis molecularly characterizes covalent modifications of DNA isolated from various sample types. Recently, the development of a high-resolution/accurate-mass data dependent approach monitoring the neutral loss of the 2′-deoxyribose moiety (116.0474 amu) and subsequent MS^3^ data acquisition has greatly advanced the field (Balbo et al., 2014a). This general approach has been successfully optimized and applied to screen for DNA adduct formation resulting from a chemotherapeutic drug and in characterizing DNA adducts resulting from a bacterial toxin of unknown chemical structure (Stornetta et al., 2015; Wilson et al., 2019). However, this approach has not yet been applied to the screening and relative quantification of endogenously generated DNA adducts. Endogenous DNA adducts are difficult to detect due to: (i) their presence at very low levels; (ii) the efficient repair systems present in cells; (iii) their wide range of polarity, which varies from hydrophilic adducts generated by ROS and aldehydes, to hydrophobic adducts, such as HNE-dG (Figure 2), generated from LPO (Swenberg et al., 2011). So far these modifications have been measured either using ^32^P-postlabelling/TLC approaches unable to specifically identify adducts or targeted LC-MS methods focusing on few modifications at a time (Beach and Gupta, 1992; Andrews et al., 1999; Chung et al., 2000; Koc and Swenberg, 2002; Singh and Farmer, 2006; Klaene et al., 2013). Profiling of multiple DNA adducts have been limited by the lack of specifically developed analytical methods and the lack of comprehensive databases.

Lists of DNA adducts have previously been reported only for adducts related to LPO and exposure to heterocyclic aromatic amines, aromatic amine and polycyclic aromatic hydrocarbon, (Hemeryck et al., 2015; Guo et al., 2017). In this project, a database containing the majority of endogenous DNA adducts reported in the literature has been created to populate an inclusion list for our method, and filling a critical gap in the field of DNA adduct analysis. This database (Table S3) is available electronically in a spreadsheet format at https://drive.google.com/open?id=14r9mA8NlL908piFCLA5yP-BZAsilxUl7. In addition to the development of the database, the sample work-up and LC-MS analysis for endogenous DNA adduct detection were optimized to comprehensively screen for the majority of endogenous DNA adducts present in mice lung tissue. In this context, the background signal, matrix effect and instrumental parameters were evaluated.

The level of background ion signal plays a central role in determining the sensitivity of LC-MS analyses, in particular for screening assays, by negatively impacting the overall analytical performance due to the finite intra-scan dynamic range and possible coalescence and self-bunching effects (Kaufmann and Walker, 2017, 2018). In the context of our methodology, an Orbitrap detector has a finite ion capacity and therefore the length of time (ion injection time) ions are collected for analysis is tightly regulated by setting a target ion number (automated gain control setting). High background ion signal in the analysis range shortens the ion injection times, reducing the number of ions of the low-level analytes, which can be collected for Orbitrap detection. This phenomenon increases the LOD of the analytes of interest. Additionally, for a data dependent approach, the selection of background ion signal for MS^2^ fragmentation reduces the time available for MS^2^ sampling of the low-level analyte ions, thus increasing the LOD. Lastly, the presence of background ion signal has the potential to reduce the ionization efficiency due to the phenomenon of ion suppression (Annesley, 2003). For these reasons, reducing the background ion signal will improve the method's ability to detect and quantify trace level DNA adducts. The comparison of the TIC signals among all the samples suggested that DNase and ALP enzymes were the main contributors to the overall background noise. We believe that when the commercial vendors purify these enzymes for sale their primary consideration is the activities of enzymes, and this results in products which are highly contaminated with various ionizable small molecule compounds. Thus, the reduction of the amount of enzymes used for the hydrolysis and their cleanliness are key factors for increasing the sensitivity of our analytical method. DNase and ALP were replaced with highly active recombinant versions allowing complete DNA digestions with lower amounts of enzymes and resulted in lower background signal, which decreased from a total ion current of 10^9^–10^8^ (Figure S6).

Adducts formed from the reaction of aldehydes with the exocyclic amines of the DNA bases generate Schiff bases which are stable in the DNA double helix, but can easily degrade once the DNA is hydrolyzed (Wang et al., 2000). The typical approach for the LC-MS analysis of Schiff base adducts is through their stabilization by reduction with a large excess of NaBH_3_CN added to the hydrolysis enzymes (Balbo et al., 2012). However, data show that NaBH_3_CN deactivates enzymes, resulting in the need for relatively large amounts of enzymes for a complete hydrolysis. It was found that removal of the NaBH_3_CN before the DNA hydrolysis allowed for the use of lower levels of enzymes and minimized background noise (Figure S1).

Another major source of background ion signal are the unmodified deoxyribonucleosides (dC, dG, T, and dA), which are present at levels many orders of magnitude higher than the modified ones, have similar masses and behave chromatographically similarly to many of the endogenous DNA adducts (Guo et al., 2017). For this reason, excluding them from the sample prior to analysis was critical to attain high sensitivity primarily for the hydrophilic adducts, and was performed by fraction collection following the developed protocol as illustrated in Figure S3. The use of HPLC fraction collection also eliminates the need for the commonly used solid-phase extraction, which have been observed to be a source of significant levels of byproducts of plastic production, such as small oligomers of polyethylene glycol, which readily ionize producing short injection times and suppression of the ionization of DNA adduct analytes (unpublished results) (Balbo et al., 2014a).

DNA adduct recovery (28–59%) after sample preparation was incomplete due to several possible factors: (1) the stability of the DNA adducts during sample work-up is unknown and decomposition could be occurring, (2) after fraction collection, a large volume of liquid is dried and reconstituted in a much smaller volume with transfer to a smaller vial, which could lead to loss of analytes, (3) during the fraction collection process, the unmodified nucleosides are purposefully removed and some adducts could partially co-chromatograph leading to their partial loss, (4) the samples are reconstituted in acetonitrile for transfer and later reconstituted in water for LC-MS analysis, which could lead to loss of some adducts due to incomplete solubilization in acetonitrile or water.

Method sensitivity was further improved by optimizing the S-Lens setting, HCD and CID collision energies and determining the relative ion trap and quadrupole isolation efficiencies as a function of isolation width. The data collected demonstrated higher ion transmission for the quadrupole at all the isolation widths tested. In addition, an S-Lens setting of 60% provided optimal transmissions of the considered adducts. The CID was set at 30% for the loss of the deoxyribose moiety and HCD at 50% for the further MS^3^ fragmentation (Figure 1). This optimized method, together with the developed sample work-up, was validated by analyzing calf thymus DNA with various amounts of the added DNA adduct standards. The method was typically able to detect 1 DNA adduct in 10^8^ unmodified nucleotides (Tables 2, 3), with a DNA adduct recovery between 28 and 59%. The instrumental method was also stable, having a standard AUC CV <10% when injecting the standard mix multiple times (Table S4). We explored our method's performance using samples from a lung carcinogenesis A/J mouse model (Melkamu et al., 2013). The main goal of this analysis was to demonstrate the method's ability to profile and characterize DNA damage of *in vivo* samples, which have undergone different exposures. In the model, exposure to the tobacco specific nitrosamine NNK induces lung tumors (Hecht et al., 1994). The NNK effect is enhanced by co-exposure with a strong pro-inflammatory agent LPS (Melkamu et al., 2013). Chronic intranasal instillation of LPS to NNK-treated mice significantly increases the multiplicity of lung tumors, and histopathologically advanced lesions (adenoma with dysplasia and adenocarcinoma); in addition to increasing macrophage recruitment to the peritumoral area, and expression of inflammation-, cell proliferation-, and survival-related proteins. NNK is a potent lung carcinogen which exerts its effects after metabolic activation known to result in the formation of various DNA adducts (Hecht and Hoffmann, 1988; Hecht, 1998, 1999). NNK is activated by cytochrome P450 enzymes, to form highly reactive species capable of generating DNA adducts. Inflammatory reactions *in vivo* involve the production and release of a range of signaling molecules including cytokines and chemokines (Grivennikov et al., 2010; Schwarze et al., 2013). *In vitro* experiments have shown that cytokines like tumor necrosis factor- α (TNF- α) formed after environmental exposures can alter the expression of metabolic enzymes such as CYPs involved in NNK bioactivation, supporting the idea that inflammation may modulate the DNA damaging activity of NNK. Additionally, LPS is known to induce various endogenous DNA adducts resulting from the generation of reactive oxygen species (ROS) and lipid peroxidation products (LPP) that may increase the overall DNA damage burden (Wu et al., 2004; Melkamu et al., 2013; Zhong and Yin, 2015). A recent study, focusing on the role of inflammation in BaP-induced DNA damage, showed by using ^32^P-Postlabelling methodology that LPS-induced pulmonary inflammation increased the amount of DNA adducts (Arlt et al., 2015). Therefore, this model offered a unique opportunity to test the ability of our method to detect numerous and chemically diverse DNA adducts.

The use of our method for the analysis of biological relevant samples demonstrated its ability to profile and chemically characterize multiple DNA adducts, and their possible isomers, generated by ROS and LPO, in addition to other exogenous exposures. The chemical characterization of DNA damage has the potential to allow for the determination of different sources of DNA damage. As mentioned in the Results section, the evaluation of the toxicological relevance of this experiment is beyond the scope of this report and will be the focus of future work.

In conclusion, the DDA-CNL/MS^3^ DNA adductomic methodology has been optimized for the screening of endogenous DNA adducts. This effort included the establishment of a publically available database of the majority of the endogenous DNA adducts reported in the literature. In addition, the sample work-up and various mass spectrometric instrumental parameters have been optimized to maximize sensitivity including: (i) use of recombinant enzymes, (ii) minimization of the amount of enzymes by removal of the reducing agent prior to DNA hydrolysis, and (iii) elimination of unmodified nucleosides via off-line fraction collection. The developed method was tested on A/J mice model and was able to detect 34 endogenous adducts, as well as NNK-derived DNA adducts in samples from mice treated with NNK. The detected DNA adducts where then targeted for PRM-MS^2^ detection and relative quantitation with three technical replicate measurements in mouse lung samples. Good precision was found for these measurements, demonstrating a robust workflow for screening and relative quantitation of large numbers of endogenous DNA adducts.

DNA adductomics, like other LC-MS-based “-omics” methodologies, is undergoing continual refinement and improvement, which is necessary to meet the analytical challenges of measuring covalent DNA damage in a comprehensive and sensitive manner (Villalta and Balbo, 2017). In the context of the present study, it may be possible to simplify data analysis by confirmation of adduct identification and elimination of MS^3^ triggered-false positives by introducing an exclusion criteria based upon an assumption that all DNA adducts must show MS^3^ signal with the appearance of nucleobases or signals diagnostic of base ion fragmentations (e.g., [Gua+H]^+^ 152.0567 *m/z* or [Gua-H_2_O]^+^ 135.1535 *m/z* for dG adducts). Another improvement in data analysis of the approach presented here would be to add the MS^2^ and MS^3^ fragmentation spectra to the endogenous DNA adduct database reported here to allow for automated confirmation of adduct identity. In addition, the sensitivity of the overall methodology could be improved through the addition of gas phase fractionation to the methodology, whereby the single full scan event is broken up into multiple ranges, allowing for greater ion injection times and enhanced ability to detect trace level DNA adducts or to measure endogenous DNA adducts using less DNA (Nazari and Muddiman, 2016). Another possible approach to increase the method sensitivity would include multiple injections of the same sample to create exclusion lists based on previous injections to allow for the detection of lower level DNA adducts with each subsequent injection (Koelmel et al., 2017). Finally, orthogonal chromatographic methods such as HILIC could be optimized for both off-line fraction collection and LC-MS to further increase the DNA adduct sensitivity and coverage.

## Data Availability Statement

The raw data supporting the conclusions of this manuscript will be made available by the authors, without undue reservation, to any qualified researcher.

## Ethics Statement

The animal study was reviewed and approved by the University of Minnesota Institutional Animal Care and Use Committee (Protocol ID: 1602-33469A) and performed in accordance with NIH guidelines.

## Author Contributions

AC and SB conceived of the idea of the manuscript and designed the experiments. FK performed all the animal experiments. PU synthetized the labeled and unlabeled DNA adduct standards. AC, VG, and RD performed the experiments and data analysis with PV's contribution. PV, SB, and VG took the lead in writing the manuscript. SB and PV supervised the project.

### Conflict of Interest

The authors declare that the research was conducted in the absence of any commercial or financial relationships that could be construed as a potential conflict of interest.
