# Supplementary material for: Targeted High Resolution LC/MS3 Adductomics Method for the Characterization of Endogenous DNA Damage
Source: Front Chem. 2019 Oct 24;7:658. doi: 10.3389/fchem.2019.00658 (PMC6822301; doi:10.3389/fchem.2019.00658)
Supplement: Supplementary file 1 [file Data_Sheet_1.docx]

Supporting Information

Targeted High Resolution LC/MS^3^ Adductomics Method for the Characterization of Endogenous DNA Damage

Andrea Carrà, Valeria Guidolin, Romel P. Dator, Pramod Upadhyaya, Fekadu Kassie, Peter W. Villalta* and Silvia Balbo*

Masonic Cancer Center, University of Minnesota, Minneapolis, MN, United States

*Correspondence:

Peter W. Villalta

villa001@umn.edu

Silvia Balbo

balbo006@umn.edu

**Table S1.** A/J mice LPS/NNK treatment timeline

|  | **Treatment** | | | |
| --- | --- | --- | --- | --- |
| **Group** | **Day-1** | **Day-2** | **Day-3** | **Day-4** |
| **1** | NNK |  |  | Sample collection |
| **2** | NNK | LPS | LPS | Sample collection |
| **3** | LPS | LPS | LPS | Sample collection |
| **4** | Vehicle | Vehicle | Vehicle | Sample collection |

**Table S2.** Experimental conditions of the DNA digestion protocols 1 and 2

| **Protocol** | **Step 1** | | **Step 2** | |
| --- | --- | --- | --- | --- |
| **1** | NaBH_3_CN+DNase+PDE-1+ALP | RT, 48h |  |  |
| **2** | NaBH_3_CN | RT, 24h | DNase+PDE-1+ALP | RT, 48h |

**LC/UV dG quantification**

Two DNA digestion protocols (Table S2) were tested using 100 ug aliquots of DNA and various amounts of hydrolysis enzymes with efficiency determined by quantitation of dG. The results, reported in Figure S1 Panel A, demonstrate that NaBH_3_CN deactivates the enzymes, thus limiting the extent of DNA hydrolysis. In the absence of NaBH_3_CN, the hydrolysis was quantitative even with relatively low levels of enzymes (protocol 2). The dG quantitation (Figure S1 Panel B) was performed by HPLC with UV detection (λ=254 nm and 10 Hz) and a C18 column (0.3 x 100 mm, 100 Ǻ, 2 um Acclaim-Thermo Scientific, Waltham, MA) operating at 40°C with a flow rate of 5 uL/min and H_2_O and CH_3_OH as mobile phases A and B, respectively. One uL of sample was injected and the following elution program was used: 2% B isocratic hold for 5min followed by a linear gradient of 1.5% B/min for 25min and a second isocratic step at 95% B for 5min. The LC-system was equilibrated at 2% CH_3_OH for 20 min prior to each injection.


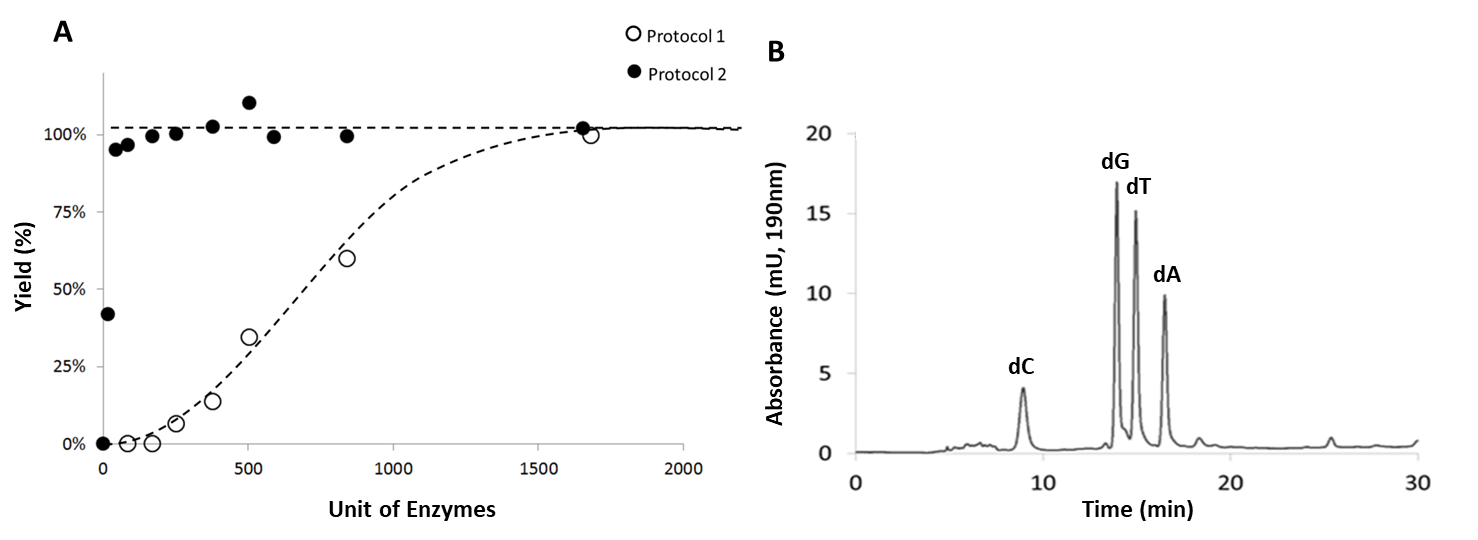


**Figure S1.** Panel A: Unit of enzymes versus digestion yield using protocols 1 and 2. Panel B: Representative chromatogram of a DNA-sample quantitatively digested enzymatically.


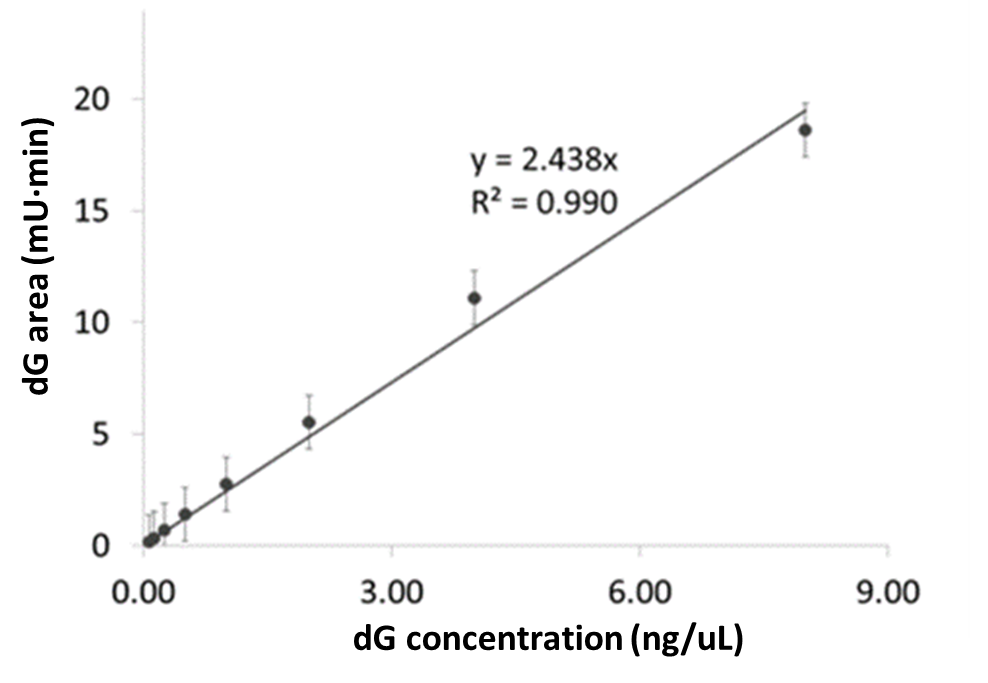


**Figure S2.** Calibration curve for dG quantification for determination of the amount of DNA digested.

The dG quantitation method had a linear response across a dynamic range of two orders of magnitude (0.125, 0.25, 0.50, 1.0, 2.0, 4.0 and 8.0 ng/uL). The limit of detection (LOD=0.04 ng/uL) was assessed by spiking decreasing amounts of dG in water and calculating the concentration required to give a S/N ratio equal to 3. The stability of the method was assessed by injecting the same calibration curve on three separate days. The coefficient of variation was found to be lower than 5%.

**Role of fraction collection in LC/MS detection**

Matrix and sample complexity play a key role in LC/MS detection. For successful trace level analyses, it is often necessary to perform significant cleanup of the sample prior to injection and this was done using fraction collection to eliminate unmodified nucleosides from the sample. Fraction collection was carried out with an HPLC (Ultimate 3000, Thermo Scientific, Waltham, MA) equipped with a C18 column (4.6 x 250 mm, 100Ǻ, 5 um Luna Phenomenex, Torrance, CA) operating at 5°C, with a flow rate of 0.5 mL/min. The mobile phases were H_2_O and CH_3_OH. The elution program involved an isocratic step at 2% CH_3_OH (5 min), followed by a linear gradient of 0.7%/min (25 min, CH_3_OH) and a second isocratic step at 100% of CH_3_OH (15 min). Before each injection, the LC system was equilibrated at isocratic conditions (2% CH_3_OH) for min 20. The mobile phase gradient and elution profile of the nucleobases were monitored by measuring two different wavelengths (λ_1_ 190 nm and λ_2_ 254 nm) at 4Hz in absorbance mode. The protocol was optimized using λ_1_ absorbance to properly fractionate the gradient, as reported in Figure S3.


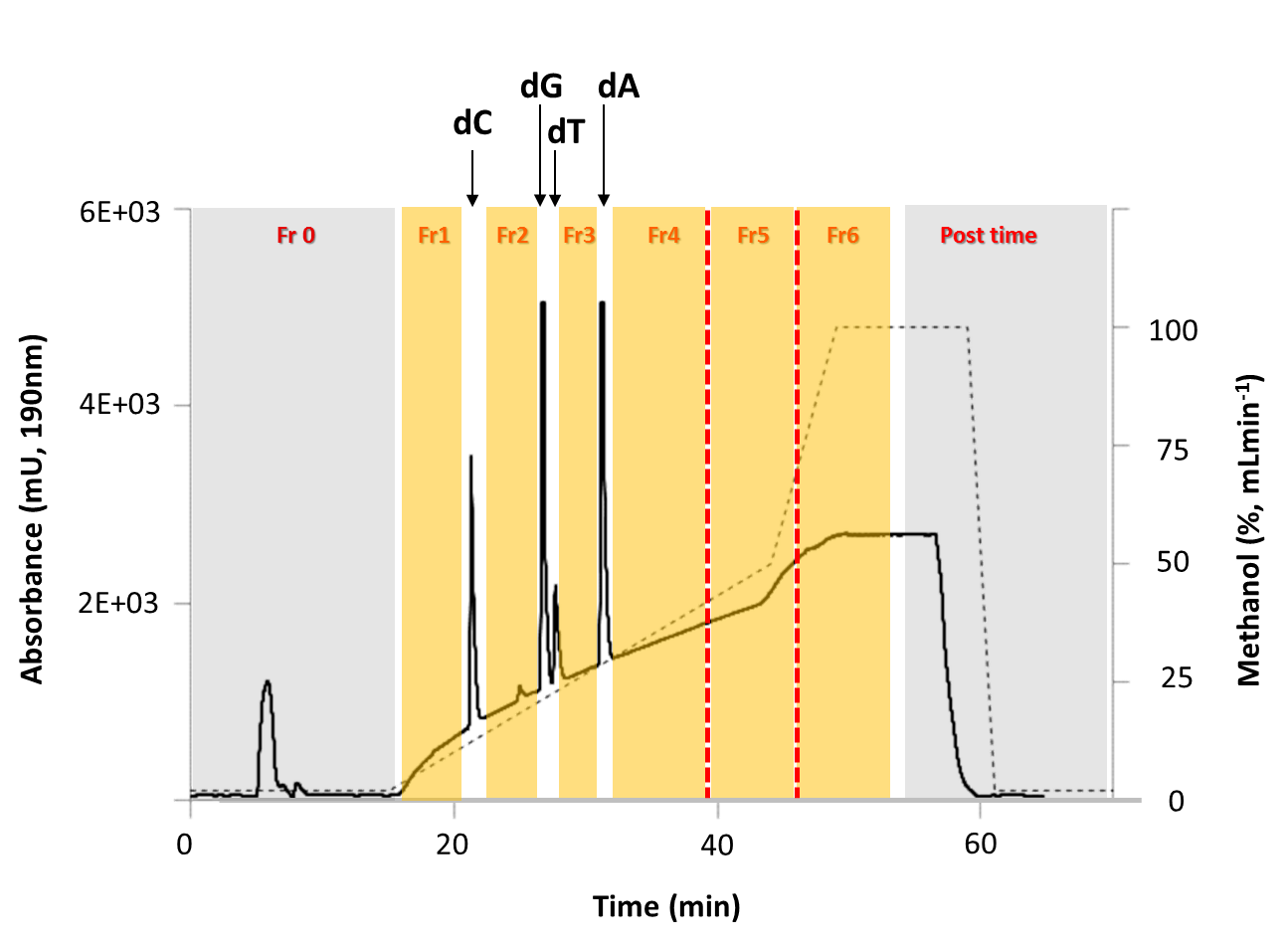


**Figure S3.** Fraction collection program with the UV chromatogram (bold line) recorded at 190 nm. The dotted line is the mobile phase gradient, yellow boxes are the collected fractions and the grey boxes are the discarded portions of the run.

**Figure S4.** Summary of DNA adducts used for method optimization and validation.


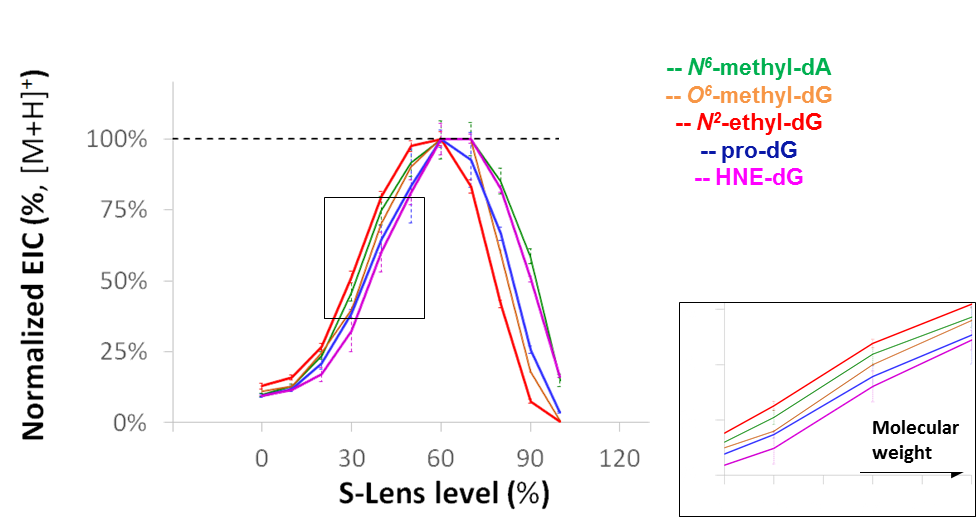


**Figure S5.** Parent ion signal intensity as a function of S-Lens setting for the infusion of five DNA adduct standards (green, brown, red, blue and violet curves for *N^6^*-methyl-dA, *O^6^*-methyl-dG, *N^2^*-ethyl-dG, prodG and HNE-dG, respectively).

**Background noise quantification**

Ion signals during DNA adduct analysis are typically very low due to the trace levels of DNA adducts, and therefore the minimization of background ion signal is critical for their successful detection and/or quantitation. To investigate ways to minimize background ion signal in our sample preparation, we quantified the contribution of each matrix component by examining the total ion chromatograms (TICs) (Figure S6). The data indicate that the DNase and ALP hydrolysis enzymes were major contributors to the overall background ion signal. Moreover, an additive effect due to all the matrix components was clearly observed.


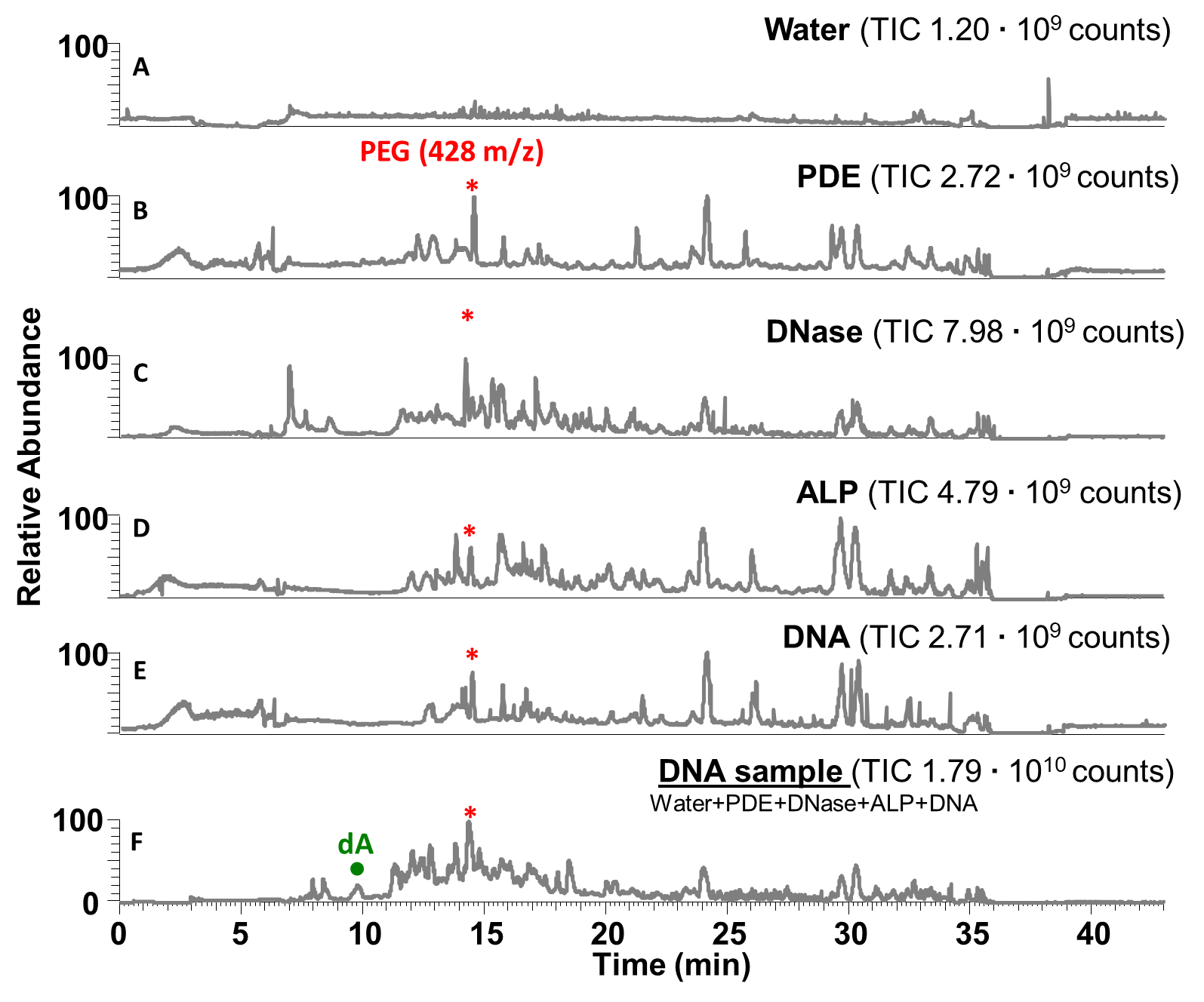


**Figure S6.** TIC chromatograms evaluating the different sources of background ion signal. Chromatogram A, B, C and D refer to water, PDE, DNase and ALP, respectively. Chromatograms E and F are the TIC traces of undigested and digested DNA samples. The asterisks (*) identified the chromatographic peak of PEG-400, an impurity common to all sample. The dots (●) identified adenine, a residual impurity originating from the enzymatic digestion of the DNA.

## Sensitivity and Linearity Measurements

## Sensitivity and detection limits were assessed by spiking different amounts of the standards into a surrogate DNA matrix and the DNA was digested using protocol 2. The purified reaction medium was divided in five aliquots of 50 µg each (0.033µmol dG) and each of those was dried and reconstituted with 20µL of isotopically labeled standard solutions containing [^15^N_5_]-*N^6^*-methyl-dA, [^15^N_5_]-*N^2^*-ethyl-dG, [^15^N_5_]-pro-dG, [^2^H_4_]-*O^2^*-POB-dT and [^2^H_4_]-*O^6^*-POB-dG (Figure S4) with each analyte at concentrations of 0.0, 0.2, 1.0, 2.0, 5.0 fmolµL^-1^. The calibration curves (Figure S7) were obtained by plotting the signals intensities of the [M+H]^+^ and [M-dR+H]^+^ ions detected with MS^1^ and MS^2^ data acquisitions, respectively.


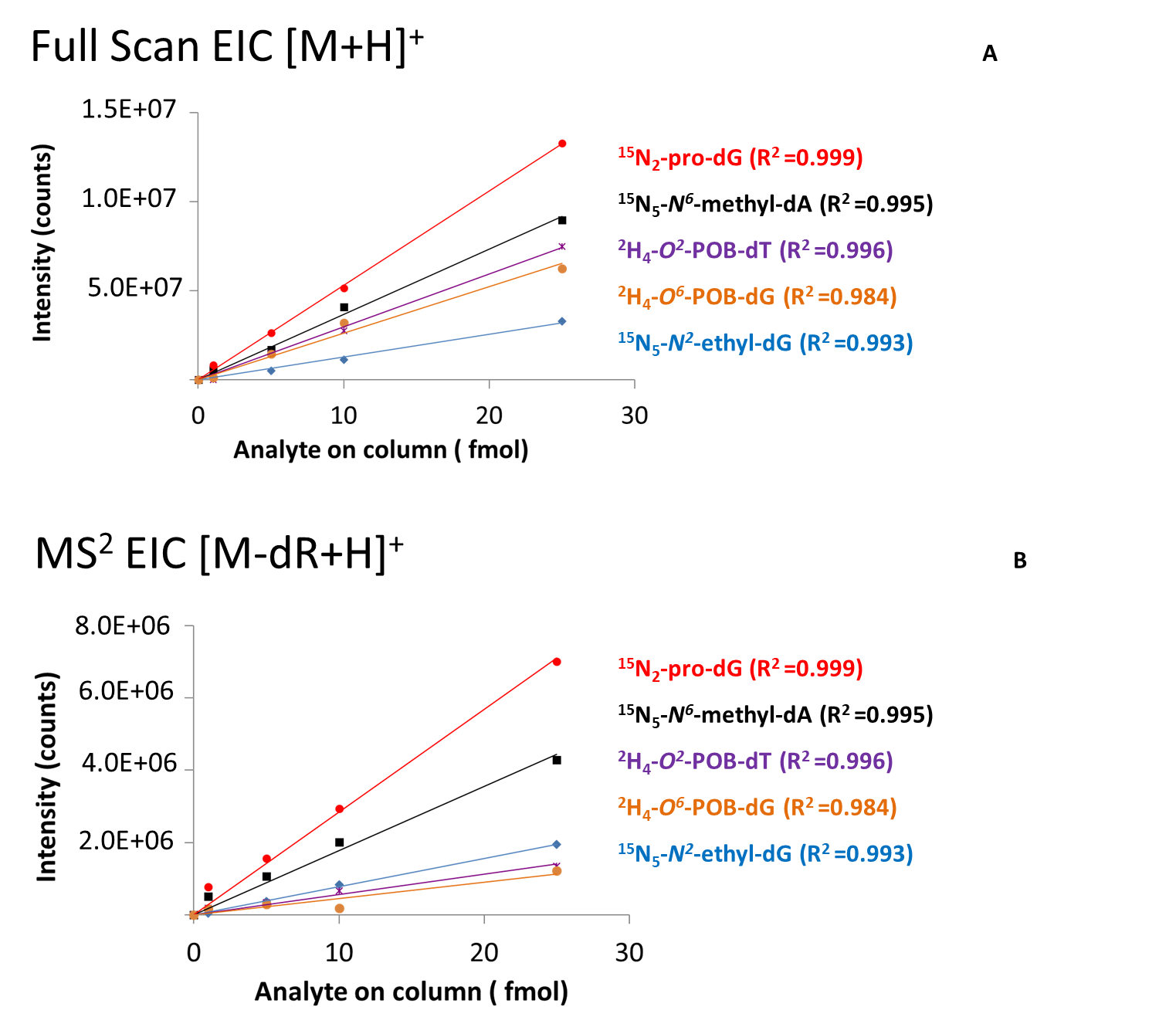


**Figure S7.** Calibration curves. Panels A and B refer to MS^1^ and MS^2^ data acquisitions, respectively.


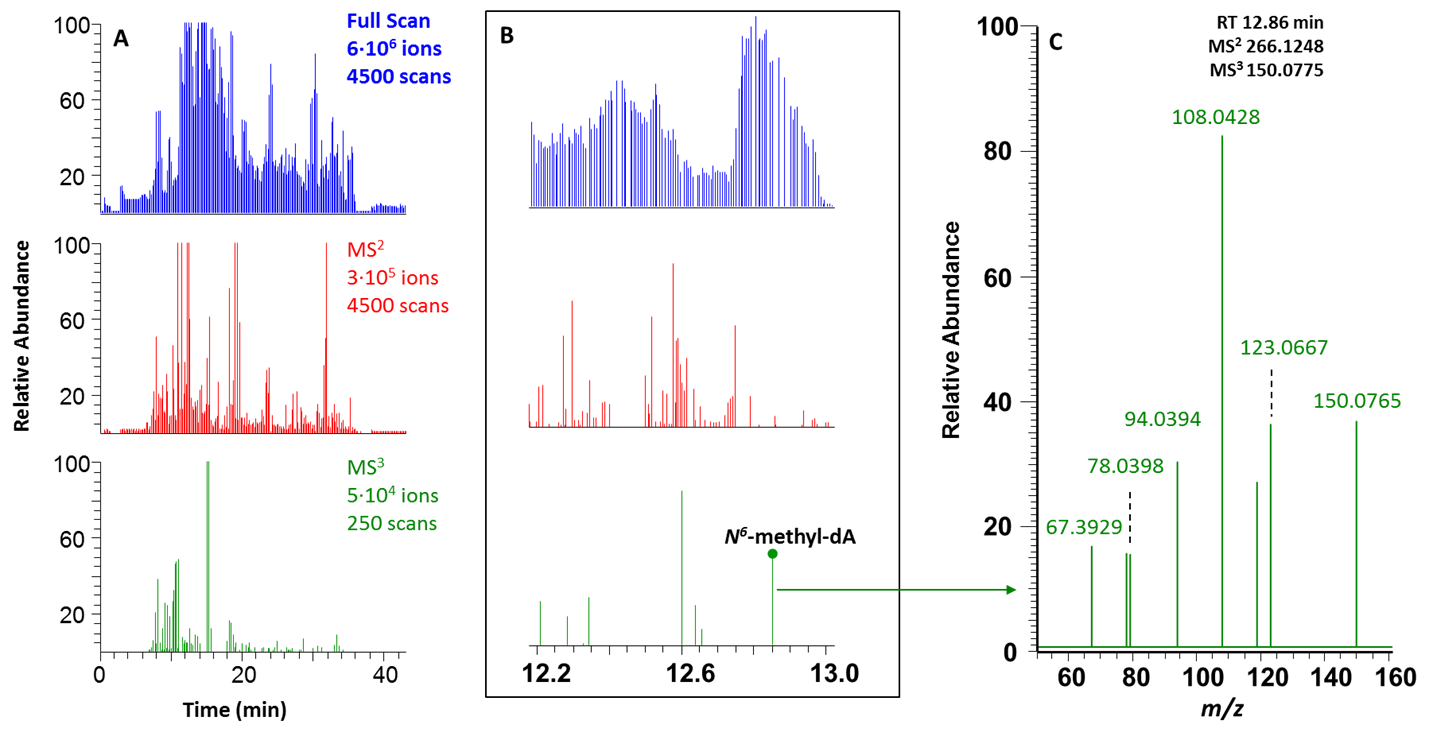


**Figure S8**. Panel A: Full Scan (blue), MS^2^ (red) and MS^3^(green) TIC chromatograms (0-43 min). Panel B: Full Scan (blue), MS^2^ (red) and MS^3^(green) TIC chromatograms (12.2-13.0 min). Panel C: MS^3^ spectrum recorded at 12.86 min. The spectrum originated from HCD fragmentation of the MS^2^ parent ion 150.0775 m/z, generated by the CID fragmentation of the precursor ion 266.1248.

The histogram in Figure S9 summarizes the number of DNA adducts detected by the adductomic approach. Only ions which triggered an MS^3^ were taken into account. EICs of the parent ions corresponding to the MS^3^ triggered events were generated from the full scan data to confirm presence and absence of the DNA adducts. Higher numbers of DNA adducts were measured in the AJ-mice exposed to both proinflammatory agent (LPS) and tobacco specific nitrosamine (NNK) as compared to the control animals. More interestingly, the synergistic effect of NNK and LPS generated more DNA adducts than from the individual exposures.


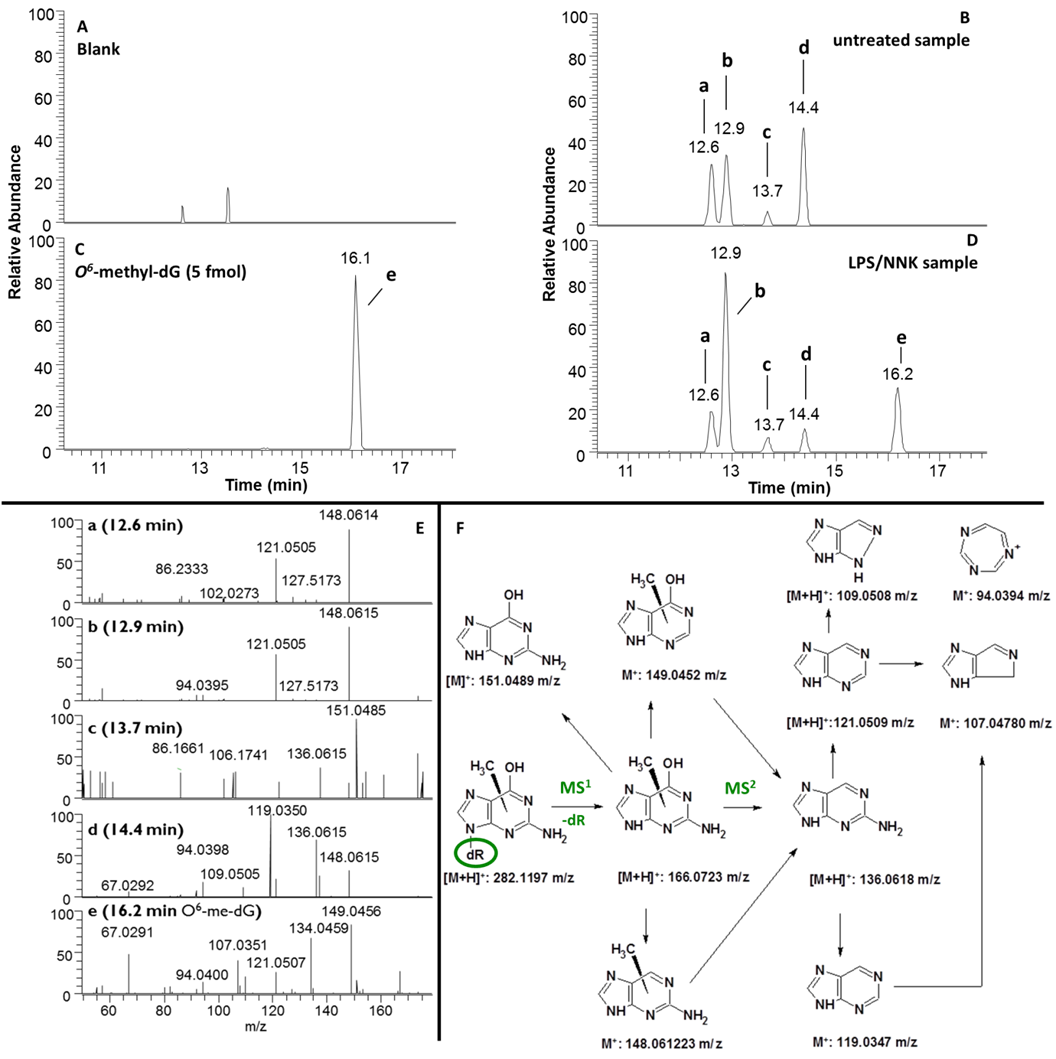


**Figure S9.** Shown are EICs for 282.1197 *m/z* in a blank sample **(A)**, a standard solution of *O^6^*-methyl-dG (2.5 fmolµL^-1^) **(C)**, DNA from untreated **(B)**, and NNK/LPS treated samples **(D)**. **B** shows EIC chromatographic peaks **a**, **b**, **c** and **d** corresponding to unknown putative DNA adducts isobaric with *O^6^*-methyl-dG. **C** shows the EIC and peak **e** for the *O^6^*-methyl-dG synthetic standard. **E** summarizes the MS^3^ spectra for the *O^6^*-methyl-dG synthetic standard **(e)** and the other unknown putative DNA adducts **(a-d)**. **F** is a proposed fragmentation pathway for the observed MS^2^and MS^3^ spectra.

**Table S3.** Endogenous DNA adduct database (available online: https://drive.google.com/open?id=14r9mA8NlL908piFCLA5yP-BZAsilxUl7)

| **Adduct Name** | **Origin** | **Chemical Formula** | **[M+H]^+^** | **Reference** | **Chemical structure** |  |
| --- | --- | --- | --- | --- | --- | --- |
|  |  |  | (*m/z*) |  |  | |
| 2,6-oxo-M_1_dG | Malonaldehyde | C_13_H_13_N_5_O_6_ | 336.0939 | (Chan and Dedon, 2010) |  | |
| 2'-Deoxyxanthosine | Oxidative stress | C_10_H_12_N_4_O_5_ | 269.0880 | (Chan and Dedon, 2010) |  | |
| 2-OH-dA | Oxidative stress | C_10_H_13_N_5_O_4_ | 268.1040 | (De Bont, 2004) |  | |
| 2-oxo-1,N^2^-εdG | Etheno | C_12_H_13_N_5_O_5_ | 308.0989 | (Chan and Dedon, 2010) |  | |
| 2-oxo-heptanone-1,*N^2^*-εdG | Etheno | C_19_H_25_N_5_O_6_ | 420.1878 | (Chan and Dedon, 2010) |  | |
| 3,*N^4^*-εdC | Lipid peroxidation | C_11_H_13_N_3_O_4_ | 252.0979 | (Marnett and Burcham, 1993) |  | |
| 1,*N^6^*- εdA | Lipid peroxidation | C_12_H_13_N_5_O_3_ | 276.1091 | (Marnett and Burcham, 1993) |  | |
| *N^3^*-methyl-A | Alkylating agent | C_6_H_7_N_5_ | 150.0774 | (Marnett and Burcham, 1993) |  | |
| *N^3^*-methyl-dA | Alkylating agent | C_11_H_16_N_5_O_3_ | 267.1320 | (Marnett and Burcham, 1993) |  | |
| 4-OH-8-oxo-dG | Oxidative stress | C_10_H_13_N_5_O_6_ | 300.0939 | (Pluskota-Karwatka, 2008) |  | |
| 4-OHE1-A | Estradiol-3,4- quinone | C_23_H_25_N_5_O_3_ | 420.2030 | (De Bont, 2004) |  | |
| 4-OHE1-G | Estradiol-3,4- quinone | C_23_H_25_N_5_O_4_ | 436.1979 | (De Bont, 2004) |  | |
| 4-OHE2-A | Estradiol-3,4- quinone | C_23_H_27_N_5_O_3_ | 422.2187 | (De Bont, 2004) |  | |
| 4-OHE2-G | Estradiol-3,4- quinone | C_23_H_27_N_5_O_4_ | 438.2136 | (De Bont, 2004) |  | |
| 5,6-H-5-OH-dU | Oxidative stress | C_9_H_15_N_3_O_6_ | 262.1034 | (Pluskota-Karwatka, 2008) |  | |
| 5,6-H-5,6-OH-dU | Oxidative stress | C_9_H_14_N_2_O_7_ | 263.0874 | (Pluskota-Karwatka, 2008) |  | |
| 5,6-H-5-OH-dC | Oxidative stress | C_9_H_15_N_3_O_5_ | 246.1084 | (Pluskota-Karwatka, 2008) |  | |
| 5,6-OH-dC | Oxidative stress | C_9_H_13_N_3_O_6_ | 260.0877 | (Pluskota-Karwatka, 2008) |  | |
| 5,6-OH-dT | Oxidative stress | C_10_H_16_N_2_O_7_ | 247.0925 | (Pluskota-Karwatka, 2008) |  | |
| 5,6-OH-dU | Oxidative stress | C_9_H_12_N_2_O_7_ | 261.0717 | (Pluskota-Karwatka, 2008) |  | |
| 5-formyl-dU | Oxidative stress | C_10_H_12_N_2_O_6_ | 257.0768 | (Pluskota-Karwatka, 2008) |  | |
| 5-hydroxymethyl-dU | Oxidative stress | C_10_H_14_N_2_O_6_ | 259.0925 | (Marnett and Burcham, 1993) (Pluskota-Karwatka, 2008) |  | |
| 5-OH-5-methylhydantoin | Oxidative stress | C_9_H_14_N_2_O_6_ | 247.0925 | (Pluskota-Karwatka, 2008) |  | |
| 5-acetyl-dC | Oxidative stress | C_12_H_17_N_3_O_5_ | 284.1241 | (Chan and Dedon, 2010) |  | |
| 5-OH-dC | Oxidative stress | C_9_H_13_N_3_O_5_ | 244.0928 | (Pluskota-Karwatka, 2008) (Marnett and Burcham, 1993) |  | |
| 5-OH-hydantoin | Oxidative stress | C_8_H_12_N_2_O_6_ | 233.0768 | (Pluskota-Karwatka, 2008) |  | |
| 5-OH-dU | Oxidative stress | C_9_H_12_N_2_O_6_ | 245.0768 | (Marnett and Burcham, 1993) |  | |
| 6-OH-8-methyl-prodG | Crotonaldehyde | C_14_H_19_N_5_O_5_ | 338.1459 | (Marnett and Burcham, 1993) |  | |
| 6-OH-dT | Oxidative stress | C_10_H_16_N_2_O_6_ | 261.1081 | (Pluskota-Karwatka, 2008) |  | |
| 6-OH-pro-dG | Acrolein | C_13_H_17_N_5_O_5_ | 324.1302 | (Marnett, 2001) |  | |
| 6-oxo-M_1_dG | Malonaldehyde | C_13_H_13_N_5_O_5_ | 320.0989 | (Chan and Dedon, 2010) |  | |
| 7- methyl-dG | Alkylating agent | C_11_H_16_N_5_O_4_ | 283.1270 | (Marnett and Burcham, 1993) |  | |
| 7- methyl-G | Alkylating agent | C_6_H_7_N_5_O | 166.0723 | (Marnett and Burcham, 1993) |  | |
| 8-nitro-dG | Oxidative stress | C_10_H_12_N_6_O_6_ | 313.0891 | (Chan and Dedon, 2010) |  | |
| 8-OH-6-methyl-prodG | Crotonaldehyde | C_14_H_19_N_5_O_5_ | 338.1459 | (Pluskota-Karwatka, 2008) (Marnett and Burcham, 1993) |  | |
| 8-OH-dA | Oxidative stress | C_10_H_13_N_5_O_4_ | 268.1040 | (De Bont, 2004) |  | |
| 8-OH-dG | Oxidative stress | C_10_H_15_N_5_O_5_ | 286.1146 | (Pluskota-Karwatka, 2008) |  | |
| 8-OH-pro-dG | Malonaldehyde | C_13_H_17_N_5_O_5_ | 324.1302 | (Marnett and Burcham, 1993) (Marnett, 2001) |  | |
| 8-oxo-dA | Oxidative stress | C_10_H_13_N_5_O_4_ | 268.1040 | (Pluskota-Karwatka, 2008) |  | |
| 8-oxo-dG | Oxidative stress | C_10_H_13_N_5_O_5_ | 284.0989 | (Marnett and Burcham, 1993) |  | |
| Acr-dA_I | Acrolein | C_13_H_17_N_5_O_4_ | 308.1353 | (Pluskota-Karwatka, 2008) |  | |
| Acr-dA_II | Acrolein | C_16_H_22_N_5_O_5_ | 365.1688 | (Pluskota-Karwatka, 2008) |  | |
| Acr-dA_III | Acrolein | C_13_H_17_N_5_O_4_ | 308.1353 | (Pluskota-Karwatka, 2008) |  | |
| Acr-dA_IV | Acrolein | C_16_H_19_N_5_O_4_ | 346.1510 | (Pluskota-Karwatka, 2008; Chou et al., 2010) |  | |
| Acr-dC_I | Acrolein | C_12_H_18_N_3_O_5_ | 285.1314 | (Pluskota-Karwatka, 2008) |  | |
| Acr-dC_II | Acrolein | C_15_H_22_N_3_O_6_ | 341.1576 | (Pluskota-Karwatka, 2008) |  | |
| Acr-dT_I | Acrolein | C_13_H_18_N_2_O_6_ | 299.1238 | (Pluskota-Karwatka, 2008) |  | |
| Acr-dT_II | Acrolein | C_16_H_24_N_2_O_8_ | 373.1605 | (Pluskota-Karwatka, 2008) |  | |
| Acr-dT_III | Acrolein | C_16_H_22_N_2_O_7_ | 355.1500 | (Pluskota-Karwatka, 2008) |  | |
| Acr-dT_IV | Acrolein | C_16_H_22_N_2_O_7_ | 355.1500 | (Pluskota-Karwatka, 2008) |  | |
| Acr-dT_V | Acrolein | C_16_H_20_N_2_O_6_ | 337.1394 | (Pluskota-Karwatka, 2008) |  | |
| *N*^2^- paraldol-dG-(5'-3')-thymidine | Acrolein | C_28_H_40_N_7_O_14_P | 730.2444 | (Pluskota-Karwatka, 2008) |  | |
| Acr-dG | Acrolein | C_18_H_27_N_5_O_7_ | 426.1983 | (Pluskota-Karwatka, 2008) |  | |
| Adenine Propenal | Malonaldehyde | C_8_H_7_N_5_O | 190.0723 | (Moriya et al., 1994) |  | |
| bis-dG | Oxidative stress | C_20_H_23_N_9_O_8_ | 518.1742 | (Schärer, 2005) |  | |
| Carboxy-dA | Glycation | C_13_H_17_N_5_O_5_ | 324.1302 | (Schärer, 2005) |  | |
| Carboxy-dC | Glycation | C_12_H_17_N_3_O_6_ | 300.1190 | (Schärer, 2005) |  | |
| Carboxynonanone-εdA | Etheno | C_21_H_27_N_5_O_6_ | 446.2034 | (Schärer, 2005) |  | |
| CE-dG | Glycation | C_13_H_17_N_5_O_6_ | 340.1252 | (Schärer, 2005) |  | |
| CHPG-dG | Glycation | C_14_H_19_N_5_O_7_ | 370.1357 | (Schärer, 2005) |  | |
| Cytosine propenal | Malonaldehyde | C_7_H_7_N_3_O_2_ | 166.0611 | (Chan and Dedon, 2010) |  | |
| *N*^2^- carboxymethyl-dG | Glycation | C_12_H_15_N_5_O_6_ | 326.1095 | (Chan and Dedon, 2010) |  | |
| CRO-G_II | Crotonaldehyde | C_13_H_17_N_5_O_3_ | 292.1404 | (Pluskota-Karwatka, 2008) |  | |
| CRO-G_III | Crotonaldehyde | C_9_H_11_N_5_O_2_ | 222.0986 | (Pluskota-Karwatka, 2008) |  | |
| CRO-dG_IV | Crotonaldehyde | C_14_H_19_N_5_O_5_ | 338.1459 | (Pluskota-Karwatka, 2008) |  | |
| DDE-dG | Lipid peroxidation | C_20_H_27_N_5_O_6_ | 434.2034 | (Loureiro et al., 2000) |  | |
| DDE-I-dA | Lipid peroxidation | C_20_H_27_N_5_O_5_ | 418.208 | (Carvalho et al., 1998) |  | |
| DDE-II-dA | Lipid peroxidation | C_20_H_29_N_5_O_6_ | 436.2191 | (Carvalho et al., 1998) |  | |
| DODE-dG | Lipid peroxidation | C_22_H_29_N_5_O_7_ | 476.2140 | (Williams et al., 2006) |  | |
| DODE-dC | Lipid peroxidation | C_21_H_29_N_3_O_7_ | 436.2078 | (Williams et al., 2006) |  | |
| dG-ACR-dG | Acrolein | C_23_H_30_N_10_O_9_ | 591.2270 | (Pluskota-Karwatka, 2008) |  | |
| dG-ACR-dG_I | Acrolein | C_23_H_28_N_10_O_8_ | 573.2164 | (Pluskota-Karwatka, 2008) |  | |
| dG-LG | Lipid peroxidation | C_30_H_45_N_5_O_9_ | 620.3290 | (Carrier et al., 2009) |  | |
| dA-LG | Lipid peroxidation | C_30_H_46_N_5_O_8_ | 604.3341 | (Carrier et al., 2009) |  | |
| dC-LG | Lipid peroxidation | C_29_H_46_N_3_O_9_ | 580.3229 | (Carrier et al., 2009) |  | |
| dIZ | Degradation of 8-oxo-dG | C_8_H_12_N_4_O_4_ | 229.0931 | (Pluskota-Karwatka, 2008) |  | |
| dOx | Degradation of 8-oxo-dG | C_8_H_14_N_4_O_5_ | 247.1037 | (Pluskota-Karwatka, 2008) |  | |
| dThdg | Oxidative stress | C_10_H_16_N2O_7_ | 277.1030 | (Marnett and Burcham, 1993) |  | |
| Oxazolone- cyanuric acid derivative | Degradation of 8-Oxo-dG | C_8_H_11_N_3_O_6_ | 246.0721 | (Pluskota-Karwatka, 2008) |  | |
| dU | Endo RNA | C_9_H_12_N_2_O_5_ | 229.0819 | (Pluskota-Karwatka, 2008) |  | |
| dUrdg | Oxidative stress | C_9_H_14_N_2_O_7_ | 263.0874 | (Marnett and Burcham, 1993) |  | |
| 4-Hydroxy-2-heptenal-dG | Lipid peroxidation | C_17_H_25_N_5_O_6_ | 396.1878 | NA |  | |
| Hex-ProdG | Lipid peroxidation | C_16_H_23_N_5_O_5_ | 366.1772 | (Douki and Ames, 1994; Gölzer et al., 1996; Doerge et al., 1998; Stout et al., 2006) |  | |
| 1,*N*^2^-εdG | Lipid peroxidation | C_12_H_13_N_5_O_4_ | 292.1040 | (Marnett and Burcham, 1993) |  | |
| FAPY-A | Oxidative stress | C_10_H_15_N_5_O_4_ | 270.1197 | (Pluskota-Karwatka, 2008) |  | |
| FAPY-dG | Oxidative stress | C_10_H_15_N_5_O_5_ | 286.1146 | (Pluskota-Karwatka, 2008) |  | |
| Guanine Propenal | Malonaldehyde | C_8_H_7_N_5_O_2_ | 206.0673 | (Weitzman et al., 1994) |  | |
| HNE-dG | Trans-4OH-2noneal | C_19_H_29_N_5_O_6_ | 424.2191 | (Doerge et al., 1998; Pluskota-Karwatka, 2008; Kozekov et al., 2010) |  | |
| HNE-dG | Lipid peroxidation | C_19_H_27_N_5_O_6_ | 422.2034 | (Rindgen et al., 1999; Douki et al., 2004; Tang et al., 2013) |  | |
| HNE-dA | Lipid peroxidation | C_19_H_27_N_5_O_5_ | 406.2085 | (Rindgen et al., 1999; Lee et al., 2000; Douki et al., 2004) |  | |
| HNE-dC | Lipid peroxidation | C_18_H_27_N_3_O_6_ | 382.1973 | (Pollack et al., 2003) |  | |
| OHE-dG | Lipid peroxidation | C_16_H_19_N_5_O_5_ | 362.1459 | (Chou et al., 2010) |  | |
| OHE-dC | Lipid peroxidation | C_15_H_19_N_3_O_5_ | 336.1554 | (Chou et al., 2010) |  | |
| ONE-dG | Lipid peroxidation | C_19_H_25_N_5_O_5_ | 404.1928 | (Williams et al., 2006; Kozekov et al., 2010) |  | |
| ONE-dA | Lipid peroxidation | C_19_H_25_N_5_O_4_ | 388.1979 | (Rindgen et al., 1999; Lee et al., 2000; Williams et al., 2006) |  | |
| ONE-dC | Lipid peroxidation | C_18_H_25_N_3_O_5_ | 364.1867 | (Williams et al., 2006) |  | |
| M_1_AA-dC | Malonaldehyde & acetaldehyde | C_14_H_17_N_3_O_5_ | 308.1241 | (Pluskota-Karwatka, 2008) |  | |
| M_1_AA-dA | Malonaldehyde & acetaldehyde | C_15_H_19_N_5_O_4_ | 334.1510 | (Pluskota-Karwatka, 2008) |  | |
| M_1_bis-dG | Malonaldehyde | C_23_H_26_N_10_O_8_ | 571.2008 | (Schärer, 2005) |  | |
| M_1_dA | Malonaldehyde | C_13_H_15_N_5_O_4_ | 306.1197 | (Pluskota-Karwatka, 2008) |  | |
| M_1_dC | Malonaldehyde | C_12_H_15_N_3_O_5_ | 282.1084 | (Pluskota-Karwatka, 2008) |  | |
| M_1_dG | Malonaldehyde | C_13_H_13_N_5_O_4_ | 304.1040 | (Marnett and Burcham, 1993) |  | |
| M_2_AA-dA | Malonaldehyde & acetaldehyde | C_18_H_19_N_5_O_5_ | 386.1459 | (Pluskota-Karwatka, 2008) |  | |
| M_2_AA-dC | Malonaldehyde & acetaldehyde | C_17_H_19_N_3_O_6_ | 362.1347 | (Pluskota-Karwatka, 2008) |  | |
| M_2_AA-dG_I | Malonaldehyde & acetaldehyde | C_18_H_19_N_5_O_6_ | 402.1408 | (Pluskota-Karwatka, 2008) |  | |
| M_2_AA-dG_II | Malonaldehyde & acetaldehyde | C_18_H_19_N_5_O_6_ | 402.1408 | (Pluskota-Karwatka, 2008) |  | |
| M_2_dG | Malonaldehyde | C_16_H_17_N_5_O_6_ | 376.1252 | (Pluskota-Karwatka, 2008) |  | |
| M_3_dA | Malonaldehyde | C_19_H_19_N_5_O_6_ | 414.1408 | (Pluskota-Karwatka, 2008) |  | |
| M_3_dC | Malonaldehyde | C_18_H_19_N_3_O_7_ | 390.1296 | (Pluskota-Karwatka, 2008) |  | |
| *3,N^2^*-εdG | Degradation of 8-oxo-dG | C_12_H_13_N_5_O_4_ | 292.1040 | (Pluskota-Karwatka, 2008) |  | |
| *N*^2^-ethyl-dG | Alkylating agent | C_12_H_17_N_5_O_4_ | 296.1353 | (Pluskota-Karwatka, 2008) |  | |
| *N^2^*OPdG | Malonaldehyde | C_13_H_15_N_5_O_5_ | 322.1146 | (Pluskota-Karwatka, 2008) | 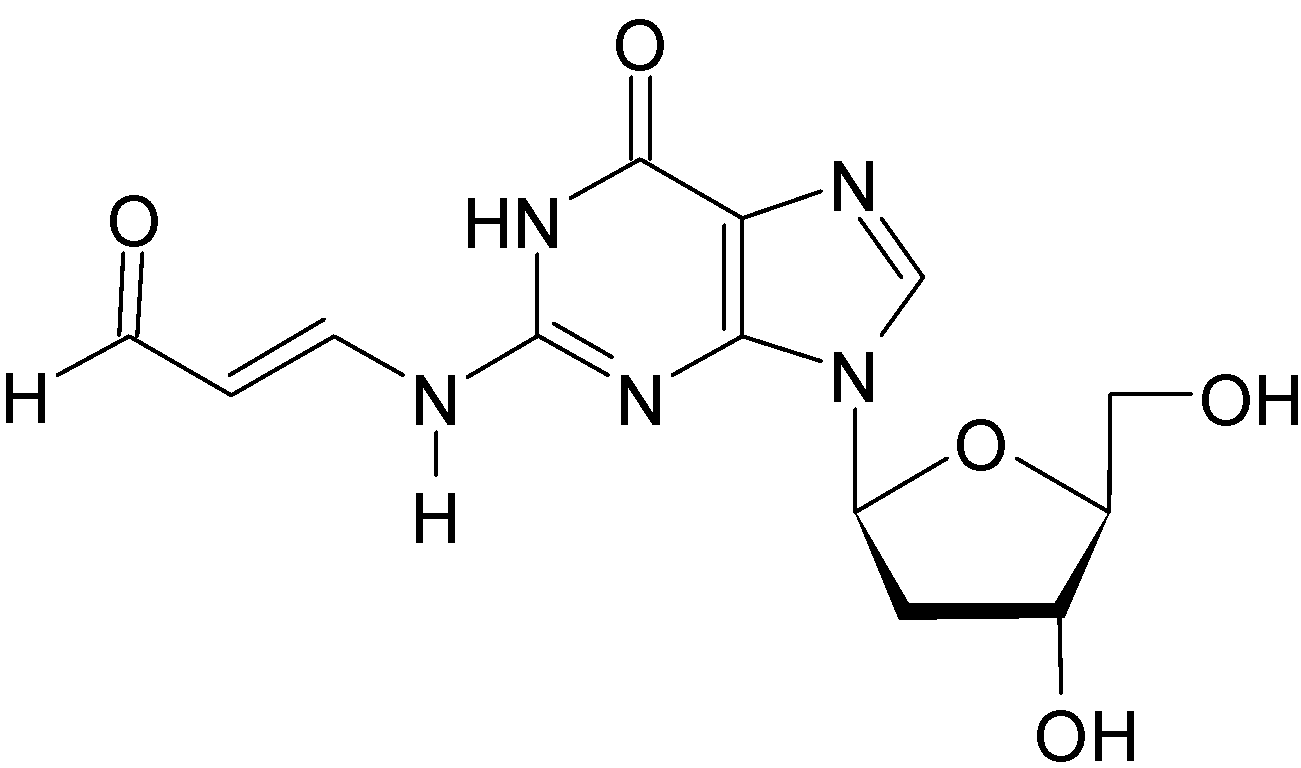 | |
| *N^2^*-paraldol-dG | Lipid peroxidation | C_16_H_23_N_5_O_6_ | 382.1721 | (Pluskota-Karwatka, 2008) |  | |
| *N^6^*-hydroxyacetyl-dA | Oxidative stress | C_12_H_15_N_5_O_5_ | 310.1146 | (Chan and Dedon, 2010) |  | |
| *N^6^*-methyl-dA | Alkylating agent | C_11_H_15_N_5_O_3_ | 266.1248 | (Chan and Dedon, 2010) |  | |
| Nitroimidazole | Oxidative stress | C_9_H_14_N_6_O_5_ | 287.1098 | (Chan and Dedon, 2010) |  | |
| Nitro-dA | Oxidative stress | C_10_H_12_N_6_O_5_ | 297.0942 | (Chan and Dedon, 2010) |  | |
| *O^4^*-methyl-dThd | Alkylating agent | C_11_H_16_N_2_O_5_ | 257.1132 | (Marnett and Burcham, 1993) |  | |
| *O^6^*-CM-dG | Glycation | C_12_H_15_N_5_O_6_ | 326.1095 | (Chan and Dedon, 2010) |  | |
| *O^6^*-methyl-dG | Alkylating agent | C_11_H_15_N_5_O_4_ | 282.1197 | (Marnett and Burcham, 1993) |  | |
| OH-1,*N^2^*-εdG | Etheno | C_12_H_15_N_5_O_6_ | 326.1095 | (Chan and Dedon, 2010) |  | |
| Oxaluric Acid | Oxidative stress | C_8_H_12_N_2_O_7_ | 249.0717 | (Chan and Dedon, 2010) |  | |
| Bicyclic oxadiazabicyclo-(3.3.0)octaimine-A | 1,4-dioxo-2-butene | C_9_H_9_N_5_O2 | 220.0829 | (Chan and Dedon, 2010) |  | |
| Bicyclic oxadiazabicyclo-(3.3.0)octaimine-C | 1,4-dioxo-2-butene | C_8_H_9_N_3_O_3_ | 196.0717 | (Chan and Dedon, 2010) |  | |
| Bicyclic oxadiazabicyclo-(3.3.0)octaimine-G_I | 1,4-dioxo-2-butene | C_9_H_9_N_5_O_3_ | 236.0778 | (Chan and Dedon, 2010) |  | |
| Bicyclic oxadiazabicyclo-(3.3.0)octaimine-G_II | 1,4-dioxo-2-butene | C_9_H_9_N_5_O_3_ | 236.0778 | (Chan and Dedon, 2010) |  | |
| Spiroiminodihydantoin | Oxidative stress | C_10_H_13_N_5_O_6_ | 300.0939 | (Chan and Dedon, 2010) |  | |
| Thymine Propenal | Malonaldehyde | C_8_H_8_N_2_O_3_ | 181.0608 | (Weitzman et al., 1994) |  | |
| Uracil-2-Propenal | Malonaldehyde | C_7_H_6_N_2_O_3_ | 167.0451 | (Weitzman et al., 1994) |  | |

## Repeatability

Instrumental repeatability was assessed by injecting a mix of standards (2.5 fmol/µL) before each experiment and comparing the instrumental responses. The statistic evaluation of the instrumental reproducibility is reported in Table S4.

**Table S4**. Statistical analysis of instrumental reproducibility

|  |  | **Assay 1** | | **Assay 2** | | **Assay 3** | | **Retention** | | |
| --- | --- | --- | --- | --- | --- | --- | --- | --- | --- | --- |
| DNA adducts | [M+H]^+^ | RT (min) | TIC | RT (min) | TIC | RT (min) | TIC | Retention | Retention | Retention |
|  |  |  |  |  |  |  |  | Avearage, min | St.Dev, min | CV, % |
| *O^6^*-ethyl-G | 180.0880 | 11.85 | 1.5E+07 | 11.85 | 1.5E+07 | 11.83 | 1.39E+07 | 11.84 | 0.01 | 0.1% |
| *N^6^* –methyl-dA | 266.1248 | 12.51 | 4.5E+06 | 12.58 | 4.2E+06 | 12.58 | 4.31E+06 | 12.56 | 0.04 | 0.3% |
| *O^2^*-POB-dT | 390.1660 | 16.60 | 2.0E+07 | 16.57 | 1.7E+07 | 16.62 | 1.84E+07 | 16.60 | 0.03 | 0.2% |
| *N*^7^-ethyl-G | 180.0880 | 8.15 | 3.5E+06 | 8.15 | 4.2E+06 | 8.19 | 3.70E+06 | 8.16 | 0.02 | 0.3% |
| Acr-dG | 324.1302 | 12.76 | 4.9E+06 | 12.74 | 5.0E+06 | 12.81 | 5.16E+06 | 12.77 | 0.04 | 0.3% |
| 1,*N^6^* –εdA | 276.1091 | 8.82 | 3.7E+07 | 8.90 | 4.1E+07 | 8.87 | 3.76E+07 | 8.86 | 0.04 | 0.5% |
| *N^2^*-ethyl-dG | 296.1353 | 15.12 | 1.2E+07 | 15.06 | 1.2E+07 | 15.14 | 1.03E+07 | 15.11 | 0.04 | 0.3% |
| *O^6^*-methyl-dG | 282.1197 | 15.04 | 8.3E+06 | 15.01 | 9.8E+06 | 15.10 | 8.60E+06 | 15.05 | 0.05 | 0.3% |
| 8-oxo-dG | 284.0989 | 12.20 | 1.5E+06 | 12.20 | 1.8E+06 | 12.17 | 1.50E+06 | 12.19 | 0.02 | 0.1% |
| HNE-dG | 424.2191 | 22.85 | 1.0E+07 | 22.87 | 1.1E+07 | 22.85 | 1.09E+07 | 22.86 | 0.01 | 0.1% |

|  |  | **Assay 1** | | **Assay 2** | | **Assay 3** | | **Detection** | | |
| --- | --- | --- | --- | --- | --- | --- | --- | --- | --- | --- |
| DNA adducts | [M+H]^+^ | RT (min) | TIC | RT (min) | TIC | RT (min) | TIC | Detection | Detection | Detection |
|  |  |  |  |  |  |  |  | Avearage, min | St.Dev, min | CV, % |
| *O^6^*-ethyl-G | 180.0880 | 11.85 | 1.5E+07 | 11.85 | 1.5E+07 | 11.83 | 1.39E+07 | 1.48E+07 | 7.84E+05 | 5% |
| *N^6^* –methyl-dA | 266.1248 | 12.51 | 4.5E+06 | 12.58 | 4.2E+06 | 12.58 | 4.31E+06 | 4.35E+06 | 1.67E+05 | 4% |
| *O^2^*-POB-dT | 390.1660 | 16.60 | 2.0E+07 | 16.57 | 1.7E+07 | 16.62 | 1.84E+07 | 1.93E+07 | 2.62E+06 | 14% |
| *N*^7^-ethyl-G | 180.0880 | 8.15 | 3.5E+06 | 8.15 | 4.2E+06 | 8.19 | 3.70E+06 | 3.81E+06 | 3.53E+05 | 9% |
| Acr-dG | 324.1302 | 12.76 | 4.9E+06 | 12.74 | 5.0E+06 | 12.81 | 5.16E+06 | 5.02E+06 | 1.41E+05 | 3% |
| 1,*N^6^* –εdA | 276.1091 | 8.82 | 3.7E+07 | 8.90 | 4.1E+07 | 8.87 | 3.76E+07 | 3.88E+07 | 2.32E+06 | 6% |
| *N^2^*-ethyl-dG | 296.1353 | 15.12 | 1.2E+07 | 15.06 | 1.2E+07 | 15.14 | 1.03E+07 | 1.16E+07 | 1.09E+06 | 9% |
| *O^6^*-methyl-dG | 282.1197 | 15.04 | 8.3E+06 | 15.01 | 9.8E+06 | 15.10 | 8.60E+06 | 8.92E+06 | 7.73E+05 | 9% |
| 8-oxo-dG | 284.0989 | 12.20 | 1.5E+06 | 12.20 | 1.8E+06 | 12.17 | 1.50E+06 | 1.53E+06 | 2.13E+05 | 14% |
| HNE-dG | 424.2191 | 22.85 | 1.0E+07 | 22.87 | 1.1E+07 | 22.85 | 1.09E+07 | 1.16E+07 | 1.92E+06 | 17% |

**Results of DNA adductomic analyses in rats trated with NNK, LPS or their combination**

**Table S5**. DNA adducts identified in lung DNA from an A/J male mice, including one control, one exposed to 100 mg/kg of NNK via intraperitoneal injection, one exposed to 5 ug of LPS by intranasal instillation and one exposed to the combination of the 2 agents. Lungs were harvested 4 days after the treatment.

| **Precursor Ions (m/z, [M+H]^+^)** | **FRG Ions (m/z, [MH-dR]^+^)** | **Retention (min)** | **Putative Identity** | **Exposure** | **Sample** |
| --- | --- | --- | --- | --- | --- |
| 310.1139 | 194.0660 | 15.04 | *N^6^*-hydroxyacetyl-dA | Oxidative stress | CTRL |
| 270.1186 | 154.0719 | 11.98 | FAPY-dA | Oxidative stress | CTRL |
| 246.1085 | 130.0607 | 7.16 | 5,6H-5-OH-dC | Oxidative stress | CTRL |
| 277.1033 | 161.0558 | 27.80 | dThdg | Oxidative stress | CTRL |
| 284.0977 | 168.0506 | 12.62 | 8-oxo-dG | Oxidative stress | CTRL |
| 286.1133 | 170.0667 | 12.42 | FAPY-dG | Oxidative stress | CTRL |
| 300.0936 | 184.0459 | 12.00 | 4-OH-8-oxo-dG | Oxidative stress | CTRL |
| 259.092 | 143.0453 | 15.44 | 5-hydroxymethyl-dU | Oxidative stress | CTRL |
| 262.1025 | 146.0558 | 7.26 | 5,6-H-5-OH-dU | Oxidative stress | CTRL |
| 268.1032 | 152.0562 | 12.08 | 2-OH-dA,8-oxo-dA | Oxidative stress | CTRL |
| 282.1070 | 166.0602 | 16.00 | *O^6^*-methyl-dG | Alkylating agent | CTRL |
| 257.1125 | 141.0652 | 13.5 | *O^4^*-methyl-dThd | Alkylating agent | CTRL |
| 266.1238 | 150.0766 | 13.80 | *N^6^-*methyl-dA | Alkylating agent | CTRL |
| 296.13400 | 180.08800 | 14.20000 | *N^2^*-ethyl-dG | Alkylating agent | CTRL |
| 417.1881 | 301.1396 | 16.98 | PHB-dG | NNK | CTRL |
| 392.1817 | 276.1334 | 17.26 | *O^2^*-PHB-dT | NNK | CTRL |
| 306.1197 | 190.0720 | 7.11 | M_1_dA | Lipid-Peroxidation | CTRL |
| 382.1719 | 266.1253 | 19 | *N^2^*-paraldol-dG | Lipid peroxidation | CTRL |
| 376.1261 | 260.0782 | 13.10 | M_2_dG | Lipid-Peroxidation | CTRL |
| 362.1332 | 246.0853 | 19.96 | M_2_AA-dC | Lipid peroxidation | CTRL |
| 334.1505 | 218.1029 | 13.35 | M_1_AA-dA | Lipid-Peroxidation | CTRL |
| 424.2183 | 308.1706 | 23.58 | HNE-dG | Lipid-Peroxidation | CTRL |
| 326.1090 | 210.0616 | 8.68 | *N*^2^-carboxymethyl-dG | Lipid-Peroxidation | CTRL |
| 340.1247 | 224.0773 | 14.28 | CE-dG | Lipid-Peroxidation | CTRL |
| 426.2196 | 310.1493 | 16.36 | ACRdT_VII | Lipid-Peroxidation | CTRL |
| 337.1385 | 221.0902 | 15 | ACRdT_V | Lipid-Peroxidation | CTRL |
| 346.1503 | 230.1020 | 19.96 | ACRdA_IV | Lipid peroxidation | CTRL |
| 324.1292 | 208.0823 | 7.63 | 6-OH-pro-dG | Lipid-Peroxidation | CTRL |
| 338.1448 | 222.0966 | 17.35 | 6-OH-8-methyl-pro-dG | Lipid-Peroxidation | CTRL |
| 308.1238 | 192.0766 | 13.73 | 2-oxo-1,N^2^-εdG | Lipid-Peroxidation | CTRL |
| 301.1201 | 185.0728 | 16.20 | [^15^N_5_]-*N^2^*-ethyl-dG | Standard | CTRL |
| 419.1974 | 303.1489 | 19.19 | [D_4_]-*O^6^*-POB-dG | Standard | CTRL |
| 421.2127 | 305.1647 | 14.78 | [D_4_]-*O^6^-*PHB-dG | Standard | CTRL |
| 394.1911 | 276.1334 | 17.60 | [D_4_]-O^2^- POB-dT | Standard | CTRL |
| 343.1309 | 227.0831 | 16.12 | [^15^N_5_]-pro-dG | Standard | CTRL |
| 310.1139 | 194.0660 | 14.23 | *N^6^*-hydroxyacetyl-dA | Oxidative stress | LPS |
| 270.1187 | 154.0717 | 11.68 | FAPY-dA | Oxidative stress | LPS |
| 246.1084 | 130.0604 | 7.09 | 5,6H-5-OH-dC | Oxidative stress | LPS |
| 277.1033 | 161.0558 | 27.8 | dThdg | Oxidative stress | LPS |
| 284.098 | 168.0510 | 11.17 | 8-oxo-dG, | Oxidative stress | LPS |
| 286.1137 | 170.0669 | 10.76 | FAPY-dG | Oxidative stress | LPS |
| 300.0932 | 184.0453 | 11.71 | 4-OH-8-oxo-dG | Oxidative stress | LPS |
| 259.092 | 143.0453 | 15.44 | 5-hydroxymethyl-dU | Oxidative stress | LPS |
| 262.1028 | 146.0554 | 7.03 | 5,6-H-5-OH-dU | Oxidative stress | LPS |
| 268.1033 | 152.0564 | 12.14 | 2-OH-dA,8-oxo-dA | Oxidative stress | LPS |
| 282.1187 | 166.0719 | 16 | *O^6^*-methyl-dG | Alkylating agent | LPS |
| 257.1125 | 141.0652 | 13.5 | *O^4^-*methyl-dThd | Alkylating agent | LPS |
| 266.1236 | 150.0769 | 13.74 | *N^6^*-methyl-dA | Alkylating agent | LPS |
| 296.1342 | 180.0876 | 15.98 | *N^2^*-ethyl-dG | Alkylating agent | LPS |
| 417.1881 | 301.1396 | 16.98 | PHB-dG | NNK | LPS |
| 392.181 | 276.1325 | 17.14 | *O^2^*-PHB-dT | NNK | LPS |
| 306.1194 | 190.0718 | 7.04 | M_1_dA | Lipid peroxidation | LPS |
| 382.1715 | 266.1239 | 18.91 | *N^2^*-paraldol-dG | Lipid peroxidation | LPS |
| 376.126 | 260.0783 | 13.49 | M_2_dG | Lipid peroxidation | LPS |
| 362.1332 | 246.0853 | 19.39 | M*_2_*AA-dC | Lipid peroxidation | LPS |
| 334.1505 | 218.1029 | 13.76 | M_1_AA-dA | Lipid-Peroxidation | LPS |
| 424.217 | 308.1703 | 25.21 | HNE-dG | Lipid-Peroxidation | LPS |
| 326.1089 | 210.0616 | 9.24 | *N*^2^-carboxymethyl-dG | Lipid-Peroxidation | LPS |
| 340.1605 | 224.1126 | 15.37 | CEG-dG | Lipid peroxidation | LPS |
| 426.1983 | 310.1493 | 21.06 | ACRdT_VII | Lipid-Peroxidation | LPS |
| 337.1385 | 221.0902 | 15.52 | ACRdT_V | Lipid-Peroxidation | LPS |
| 346.1496 | 230.1020 | 19.39 | ACRdA_IV | Lipid peroxidation | LPS |
| 324.1296 | 208.0816 | 7.65 | 6-OH-pro-dG | Lipid-Peroxidation | LPS |
| 338.145 | 222.0979 | 16.63 | 6-OH-8-methyl-pro-dG | Lipid-Peroxidation | LPS |
| 308.1236 | 192.0761 | 14.4 | 2-oxo-1,N^2^-εdG | Lipid-Peroxidation | LPS |
| 301.1203 | 185.0727 | 16.23 | [^15^N_5_]-*N^2^*-ethyl-dG | Standard | LPS |
| 419.19971 | 301.1396 | 16.98 | [D_4_]- *O^6^*-POB-dG | Standard | LPS |
| 421.2124 | 305.1649 | 15.19 | [D_4_]- *O^6^*-PHB-dG | Standard | LPS |
| 394.1906 | 278.1430 | 17.51 | [D_4_]- *O^2^-*POB-dT | Standard | LPS |
| 343.1311 | 227.0831 | 16.1 | [^15^N_5_]-prodG | Standard | LPS |
| 310.1139 | 194.0660 | 15.04 | *N^6^*-hydroxyacetyl-dA | Oxidative stress | NNK |
| 270.1186 | 154.0717 | 8.7 | FAPY-dA | Oxidative stress | NNK |
| 246.1085 | 130.0607 | 7.14 | 5,6H-5-OH-dC | Oxidative stress | NNK |
| 277.1033 | 161.0558 | 27.8 | dThdg | Oxidative stress | NNK |
| 284.0984 | 168.0510 | 12.61 | 8-oxo-dG | Oxidative stress | NNK |
| 286.1134 | 170.0663 | 12.02 | FAPY-dG | Oxidative stress | NNK |
| 300.0936 | 184.0464 | 11.74 | 4-OH-8-oxo-dG | Oxidative stress | NNK |
| 259.092 | 143.0453 | 15.44 | 5-hydroxymethyl-dU | Oxidative stress | NNK |
| 262.103 | 146.0558 | 6.99 | 5,6-H-5-OH-dU | Oxidative stress | NNK |
| 268.1032 | 152.0564 | 12.09 | 2-OH-dA,8-oxo-dA | Oxidative stress | NNK |
| 282.1186 | 166.0719 | 16.13 | *O^6^*-methyl-dG | Alkylating agent | NNK |
| 257.1125 | 141.0652 | 13.5 | *O^4^-*methyl-dThd | Alkylating agent | NNK |
| 266.1236 | 150.0769 | 13.3 | *N^6^*-methyl-dA | Alkylating agent | NNK |
| 296.134 | 180.0873 | 15.7 | *N^2^*-ethyl-dG | Alkylating agent | NNK |
| 417.1881 | 301.1396 | 16.98 | PHB-dG | NNK | NNK |
| 392.1817 | 276.1334 | 17.83 | *O^2^*-PHB-dT | NNK | NNK |
| 306.1197 | 190.0720 | 7.16 | M_1_dA | Lipid-Peroxidation | NNK |
| 382.1719 | 266.1253 | 19 | *N^2^*-paraldol-dG | Lipid peroxidation | NNK |
| 376.1261 | 260.0788 | 13.22 | M_2_dG | Lipid-Peroxidation | NNK |
| 362.1332 | 246.0853 | 19.96 | M_2_AA-dC | Lipid peroxidation | NNK |
| 334.1505 | 218.1029 | 13.76 | M_1_AA-dA | Lipid-Peroxidation | NNK |
| 424.2175 | 308.1706 | 23.8 | HNE-dG | Lipid-Peroxidation | NNK |
| 326.1097 | 210.0614 | 8.31 | *N*^2^-carboxymethyl-dG | Lipid-Peroxidation | NNK |
| 340.1251 | 224.0767 | 14.83 | CEG-dG | Lipid-Peroxidation | NNK |
| 426.21963 | 310.1493 | 16.42 | ACRdT_VII | Lipid-Peroxidation | NNK |
| 337.1385 | 221.0902 | 15 | ACRdT_V | Lipid-Peroxidation | NNK |
| 346.1503 | 230.1020 | 19.96 | ACRdA_IV | Lipid peroxidation | NNK |
| 324.1292 | 208.0822 | 7.6 | 6-OH-prodG | Lipid-Peroxidation | NNK |
| 338.1448 | 222.0966 | 17.35 | 6-OH-8-methyl-pro-dG | Lipid-Peroxidation | NNK |
| 308.1238 | 192.0766 | 13.64 | 2-oxo-1,N^2^-εdG | Lipid-Peroxidation | NNK |
| 301.1203 | 185.0727 | 16.23 | [^15^N_5_]-*N*^2^-ethyl-dG | Standard | NNK |
| 419.19971 | 301.1396 | 19.25 | [D_4_]-O^6^-POB-dG | Standard | NNK |
| 421.2148 | 305.1672 | 14.78 | [D_4_]-*O^6^*- PHB-dG | Standard | NNK |
| 394.19 | 278.1400 | 17.6 | [D_4_]-*O^2^*- POB-dT | Standard | NNK |
| 343.1299 | 227.0818 | 16.09 | [^15^N_5_]-pro-dG | Standard | NNK |
| 310.1139 | 194.0660 | 0.2115 | *N^6^*-hydroxyacetyl-dA | Oxidative stress | LPS+NNK |
| 270.1187 | 154.0717 | 0.8942 | FAPY-dA | Oxidative stress | LPS+NNK |
| 246.1084 | 130.0604 | 0.0165 | 5,6H-5-OH-dC | Oxidative stress | LPS+NNK |
| 277.1033 | 161.0558 | 0.0000 | dThdg | Oxidative stress | LPS+NNK |
| 284.0989 | 168.0842 | 2.3678 | 8-oxo-dG | Oxidative stress | LPS+NNK |
| 286.1137 | 170.0669 | 0.0000 | FAPY-dG | Oxidative stress | LPS+NNK |
| 300.0931 | 184.0462 | 0.2933 | 4-OH-8-oxo-dG | Oxidative stress | LPS+NNK |
| 259.0920 | 143.0453 | 0.0000 | 5-hydroxymethyl-dU | Oxidative stress | LPS+NNK |
| 262.1025 | 146.0558 | 0.1147 | 5,6-H-5-OH-dU | Oxidative stress | LPS+NNK |
| 268.1034 | 152.0561 | 8.9663 | 2-OH-dA,8-oxo-dA | Oxidative stress | LPS+NNK |
| 282.1187 | 166.0720 | 9.5072 | *O^6^*-methyl-dG | Alkylating agent | LPS+NNK |
| 257.1125 | 141.0652 | 0.0000 | *O^4^*-methyl-dThd | Alkylating agent | LPS+NNK |
| 266.1241 | 150.0771 | 2.8245 | *N^6^*-methyl-dA | Alkylating agent | LPS+NNK |
| 296.1435 | 180.0880 | 3.9063 | *N^2^*-ethyl-dG | Alkylating agent | LPS+NNK |
| 417.1881 | 301.1396 | 0.0000 | PHB-dG | NNK | LPS+NNK |
| 392.1808 | 276.1335 | 0.6659 | *O^2^*-PHB-dT | NNK | LPS+NNK |
| 306.1197 | 190.0722 | 0.4243 | M_1_dA | Lipid peroxidation | LPS+NNK |
| 382.1719 | 266.1253 | 0.0000 | *N^2^*-paraldol-dG | Lipid peroxidation | LPS+NNK |
| 376.1265 | 260.0783 | 0.5361 | M_2_-dG | Lipid peroxidation | LPS+NNK |
| 362.1332 | 246.0853 | 0.0000 | M_2_AA-dC | Lipid peroxidation | LPS+NNK |
| 334.1505 | 218.1029 | 1.1647 | M_1_AA-dA | Lipid-Peroxidation | LPS+NNK |
| 424.2212 | 308.1733 | 0.6550 | HNE-dG | Lipid-Peroxidation | LPS+NNK |
| 326.1097 | 210.0614 | 0.0000 | *N*^2^- carboxymethyl-dG | Lipid-Peroxidation | LPS+NNK |
| 340.1246 | 224.0771 | 1.8389 | CEG-dG | Lipid peroxidation | LPS+NNK |
| 426.2196 | 310.1493 | 0.0000 | ACRdT_VII | Lipid-Peroxidation | LPS+NNK |
| 337.1385 | 221.0902 | 0.0000 | ACRdT_V | Lipid-Peroxidation | LPS+NNK |
| 346.1496 | 230.1020 | 0.0000 | ACRdA_IV | Lipid peroxidation | LPS+NNK |
| 324.1296 | 208.0816 | 0.0791 | 6-OH-pro-dG | Lipid-Peroxidation | LPS+NNK |
| 338.1550 | 222.0979 | 0.0000 | 6-OH-8-methyl-prodG | Lipid-Peroxidation | LPS+NNK |
| 308.1239 | 192.0764 | 0.1863 | 2-oxo-1,N^2^-εdG | Lipid-Peroxidation | LPS+NNK |
| 301.1203 | 185.0727 | 2.5000 | [^15^N_5_]-*N*^2^-ethyl-dG | Standard | LPS+NNK |
| 419.1970 | 303.1494 | 1.2981 | [D_4_]-*O*^6^-POB-dG | Standard | LPS+NNK |
| 421.2127 | 305.1647 | 1.6346 | [D_4_]-*O*^6^- PHB-dG | Standard | LPS+NNK |
| 394.1902 | 278.1431 | 1.0300 | [D_4_]-*O^2^*-POB-dT | Standard | LPS+NNK |
| 343.1298 | 227.0821 | 5.1563 | [^15^N_5_]-pro-dG | Standard | LPS+NNK |

**References**

Carrier, E.J., Amarnath, V., Oates, J.A., and Boutaud, O. Characterization of Covalent Adducts of Nucleosides and DNA Formed by Reaction with Levuglandin. *Biochemistry* (2009) 48(45)**,** 10775-10781. doi: 10.1021/bi9015132.

Carvalho, V.M., Di Mascio, P., de Arruda Campos, I.P., Douki, T., Cadet, J., and Medeiros, M.H.G. Formation of 1,N6-Etheno-2‘-deoxyadenosine Adducts bytrans,trans-2,4-Decadienal. *Chemical Research in Toxicology* (1998) 11(9)**,** 1042-1047. doi: 10.1021/tx9800710.

Chan, S.W., and Dedon, P.C. The Biological and Metabolic Fates of Endogenous DNA Damage Products. *Journal of Nucleic Acids* (2010) 2010**,** 1-13. doi: 10.4061/2010/929047.

Chou, P.-H., Kageyama, S., Matsuda, S., Kanemoto, K., Sasada, Y., Oka, M., et al. Detection of Lipid Peroxidation-Induced DNA Adducts Caused by 4-Oxo-2(E)-nonenal and 4-Oxo-2(E)-hexenal in Human Autopsy Tissues. *Chemical Research in Toxicology* (2010) 23(9)**,** 1442-1448. doi: 10.1021/tx100047d.

De Bont, R. Endogenous DNA damage in humans: a review of quantitative data. *Mutagenesis* (2004) 19(3)**,** 169-185. doi: 10.1093/mutage/geh025.

Doerge, D.R., Yi, P., Churchwell, M.I., Preece, S.W., Langridge, J., and Fu, P.P. Mass spectrometric analysis of 2′-deoxyribonucleoside and 2′-deoxyribonucleotide adducts with aldehydes derived from lipid peroxidation. *Rapid Communications in Mass Spectrometry* (1998) 12(22)**,** 1665-1672. doi: 10.1002/(sici)1097-0231(19981130)12:22<1665::aid-rcm384>3.3.co;2-#.

Douki, T., and Ames, B.N. An HPLC-EC Assay for 1,N2-Propano Adducts of 2'-Deoxyguanosine with 4-Hydroxynonenal and Other .alpha.,.beta.-Unsaturated Aldehydes. *Chemical Research in Toxicology* (1994) 7(4)**,** 511-518. doi: 10.1021/tx00040a006.

Douki, T., Odin, F., Caillat, S., Favier, A., and Cadet, J. Predominance of the 1,N2-propano 2′-deoxyguanosine adduct among 4-hydroxy-2-nonenal-induced DNA lesions. *Free Radical Biology and Medicine* (2004) 37(1)**,** 62-70. doi: 10.1016/j.freeradbiomed.2004.04.013.

Gölzer, P., Janzowski, C., Pool-Zobel, B.L., and Eisenbrand, G. (E)-2-Hexenal-Induced DNA Damage and Formation of Cyclic 1,N2-(1,3-Propano)-2‘-deoxyguanosine Adducts in Mammalian Cells. *Chemical Research in Toxicology* (1996) 9(7)**,** 1207-1213. doi: 10.1021/tx9600107.

Kozekov, I.D., Turesky, R.J., Alas, G.R., Harris, C.M., Harris, T.M., and Rizzo, C.J. Formation of Deoxyguanosine Cross-Links from Calf Thymus DNA Treated with Acrolein and 4-Hydroxy-2-nonenal. *Chemical Research in Toxicology* (2010) 23(11)**,** 1701-1713. doi: 10.1021/tx100179g.

Lee, S.H., Rindgen, D., Bible, R.H., Hajdu, E., and Blair, I.A. Characterization of 2‘-Deoxyadenosine Adducts Derived from 4-Oxo-2-nonenal, a Novel Product of Lipid Peroxidation. *Chemical Research in Toxicology* (2000) 13(7)**,** 565-574. doi: 10.1021/tx000057z.

Loureiro, A.P.M., Di Mascio, P., Gomes, O.F., and Medeiros, M.H.G. trans,trans-2,4-Decadienal-Induced 1,N2-Etheno-2‘-deoxyguanosine Adduct Formation. *Chemical Research in Toxicology* (2000) 13(7)**,** 601-609. doi: 10.1021/tx000004h.

Marnett, L. Endogenous DNA damage and mutation. *Trends in Genetics* (2001) 17(4)**,** 214-221. doi: 10.1016/s0168-9525(01)02239-9.

Marnett, L.J., and Burcham, P.C. Endogenous DNA adducts: Potential and paradox. *Chemical Research in Toxicology* (1993) 6(6)**,** 771-785. doi: 10.1021/tx00036a005.

Moriya, M., Zhang, W., Johnson, F., and Grollman, A.P. Mutagenic potency of exocyclic DNA adducts: marked differences between Escherichia coli and simian kidney cells. *Proceedings of the National Academy of Sciences* (1994) 91(25)**,** 11899-11903. doi: 10.1073/pnas.91.25.11899.

Pluskota-Karwatka, D. Modifications of nucleosides by endogenous mutagens–DNA adducts arising from cellular processes. *Bioorganic Chemistry* (2008) 36(4)**,** 198-213. doi: 10.1016/j.bioorg.2008.04.002.

Pollack, M., Oe, T., Lee, S.H., Silva Elipe, M.V., Arison, B.H., and Blair, I.A. Characterization of 2‘-Deoxycytidine Adducts Derived from 4-Oxo-2-nonenal, a Novel Lipid Peroxidation Product. *Chemical Research in Toxicology* (2003) 16(7)**,** 893-900. doi: 10.1021/tx030009p.

Rindgen, D., Nakajima, M., Wehrli, S., Xu, K., and Blair, I.A. Covalent Modifications to 2‘-Deoxyguanosine by 4-Oxo-2-nonenal, a Novel Product of Lipid Peroxidation. *Chemical Research in Toxicology* (1999) 12(12)**,** 1195-1204. doi: 10.1021/tx990034o.

Schärer, O.D. DNA Interstrand Crosslinks: Natural and Drug-Induced DNA Adducts that Induce Unique Cellular Responses. *ChemBioChem* (2005) 6(1)**,** 27-32. doi: 10.1002/cbic.200400287.

Stout, M.D., Jeong, Y.-C., Boysen, G., Li, Y., Sangaiah, R., Ball, L.M., et al. LC/MS/MS Method for the Quantitation oftrans-2-Hexenal-Derived Exocyclic 1,N2-Propanodeoxyguanosine in DNA. *Chemical Research in Toxicology* (2006) 19(4)**,** 563-570. doi: 10.1021/tx050346t.

Tang, Y., Kassie, F., Qian, X., Ansha, B., and Turesky, R.J. DNA Adduct Formation of 2-Amino-9H-pyrido[2,3-b]indole and 2-Amino-3,4-dimethylimidazo[4,5-f]quinoline in Mouse Liver and Extrahepatic Tissues During a Subchronic Feeding Study. *Toxicological Sciences* (2013) 133(2)**,** 248-258. doi: 10.1093/toxsci/kft077.

Weitzman, S.A., Turk, P.W., Milkowski, D.H., and Kozlowski, K. Free radical adducts induce alterations in DNA cytosine methylation. *Proceedings of the National Academy of Sciences* (1994) 91(4)**,** 1261-1264. doi: 10.1073/pnas.91.4.1261.

Williams, M.V., Lee, S.H., Pollack, M., and Blair, I.A. Endogenous Lipid Hydroperoxide-mediated DNA-adduct Formation in Min Mice. *Journal of Biological Chemistry* (2006) 281(15)**,** 10127-10133. doi: 10.1074/jbc.m600178200.
